# Decision-making about bariatric and cosmetic medical tourism from countries with universal healthcare: a rapid systematic review

**DOI:** 10.1186/s12992-026-01207-x

**Published:** 2026-03-28

**Authors:** Beth Nichol, Devashish Ray, Louise Tanner, Emily J. Oliver, Ivo Vlaev, Falko Sniehotta, Laura McGowan

**Affiliations:** 1 NIHR Policy Research Unit Behavioural and Social Sciences, Population Health Sciences Institute, Faculty of Medical Sciences, Newcastle University, Newcastle, UK; 2 School of Communities and Education, Faculty of Health and Life Sciences, Northumbria University, Newcastle, UK; 3 NIHR Innovation Observatory, Newcastle University, Newcastle upon Tyne, UK; 4 Centre for Behavioral and Implementation Science Interventions (BISI), Yong Loo Lin School of Medicine, National University of Singapore, Queenstown, Singapore; 5 Division of Prevention, Center for Preventive Medicine and Digital Health (CPD), Medical Faculty Mannheim, Heidelberg University, Mannheim, Germany

## Abstract

**Background:**

A growing number of people are travelling abroad for medical treatments (known as medical tourism; MT), driven by globalisation of the healthcare market. Medical tourists from countries with universal healthcare strain resources when returning with complications which require aftercare or further treatment. This rapid systematic review synthesised literature on decision-making of medical tourists, focusing on cosmetic and bariatric MT as high-demand and high-risk areas, to identify opportunities to support informed decision-making (PROSPERO registration number: CRD420251000992).

**Methods:**

Guided by Cochrane guidance for rapid reviews, three databases (MEDLINE^®^, PsycINFO, and Web of Science) plus grey literature (Overton) were searched on the 24th February 2025 for qualitative, quantitative, or mixed methods studies that focused on outbound cosmetic or bariatric medical tourists from countries with universal healthcare. Narrative synthesis, thematic synthesis, and triangulation were applied. The Mixed Methods Appraisal Tool (MMAT) was used to assess the quality of included studies. Public involvement shaped the search methods and data interpretation.

**Results:**

A total of 25 studies (12 qualitative, 11 quantitative, and two mixed methods) were included. Quantitative data indicated a primary motivator of cost. Thematic synthesis identified three overarching themes: (1) Medical Tourists as Constrained Consumers: Decision-Making under Systemic Healthcare Limitations, (2) Crowdsourcing Trust: Peer-Produced Evidence and Trusting Strangers in the Absence of Support, and (3) Informed but Invested: Navigating Risk Through Emotional Commitment. Triangulation identified quantitative research gaps particularly around information seeking behaviour and the effect of emotional investment on risk perception.

**Discussion:**

Medical tourists are mainly limited in their decision-making by inaccessibility of universal healthcare and, subsequently, cost of treatment. Medical tourists are emotionally invested in receiving surgery, resulting in them minimising perceived risks. Medical tourists also rely heavily on anecdotal information, especially from online communities, in a context of inaccurate information and low social support. Prospective studies with generalisable samples are needed, particularly for bariatric tourism. Limitations include the limited value of available quantitative data in understanding the decision-making process of medical tourists.

**Conclusions:**

Given the strong financial appeal of surgery abroad, efforts to reduce the harms of MT should prioritise harm reduction strategies over deterrence, which leverage the persuasive power of anecdotal evidence and strengthen communication with domestic healthcare providers.

**Supplementary Information:**

The online version contains supplementary material available at 10.1186/s12992-026-01207-x.

## Background

An increasing number of residents from high income countries such as the United Kingdom (UK) are choosing to travel abroad for medical, surgical or dental treatment [1], known as medical tourism [2] (MT). Globalisation has facilitated the ideal environment for the MT industry to flourish [3]; online interconnectivity has allowed for marketing that disseminates advertisements and information across borders, and increased physical interconnectivity translates into ease and accessibility of international travel [4]. Such conditions have created a growing and lucrative market for frequent destination countries [5]. Amongst the most common forms of MT is cosmetic surgery (a type of elective surgery focused on enhancing a person’s physical appearance for aesthetic reason), known as ‘cosmetic tourism’ [6]. MT for weight loss surgery, known as ‘bariatric tourism’ [7] is also common due to limited eligibility and long waiting lists [8]. For example, availability of weight loss surgery within the UK is limited to individuals with a BMI of *≥* 40, or between 35 and < 40 with comorbidities who can provide evidence of attempts to lose weight through non-surgical interventions [9] and tends to be offered to older patients already experiencing obesity-related disease [10]. Subsequently, within the UK, the number of medical tourists travelling abroad for bariatric surgery is estimated to be equal to or exceeding the number of patients receiving bariatric care via the NHS [11].

A number of medical tourists are returning with complications from procedures not usually covered by their home country’s universal healthcare system, seeking follow-on care [12]. Regardless of the competence of individual surgeons at medical tourism destinations, there are other risk factors for complications associated with MT, including short hospital stays, postoperative travel, and inadequate aftercare [13, 14]. Complications are most often infections following cosmetic procedures [15, 16], and anastomotic leakage (between connecting or opening sites from surgery), pulmonary embolism, gastric complications, and requirement for reversal of surgery following bariatric tourism [17]. There is a lack of systematic, national data on the true scope of MT and its associated harms [2, 12, 18], with existing research focusing on single centres [6, 12, 19–23]. For example, generalisation from a single tertiary centre in the UK estimates that cosmetic tourism costs £8.2 million to the UK NHS per annum, whilst bariatric tourism presents a more complex picture, as in some cases it may reduce the financial impact of obesity [1]. More data is needed on the net harms and associated costs of cosmetic and bariatric tourism. One available evaluation, within Alberta, Canada, reported that bariatric tourism costs the universal healthcare system $1.9 million Canadian Dollars more than bariatric patients within the universal healthcare system, when including costs of the surgery, complications, and reimbursement [24]. Although the Canadian universal healthcare system provides reimbursement to bariatric tourists receiving surgery abroad ($100 per night in hospital and/or $50 per outpatient visit), the findings suggest an increased incidence of complications and need for aftercare for bariatric tourists, such that they exceed any averted costs of the surgery itself [24]. Subsequently, domestic surgeons and General Practitioners (GPs) have called for government action [11], including international regulation [25].

Due to the international and therefore cross-border nature of MT, reducing the harms of MT through regulation is challenging due to conflicting interests between majority home versus destination countries [18]. For example, consensus European recommendations have been developed for healthcare professionals (HCPs) providing bariatric surgery [26], but how they will be enforced in a global market is unknown. Thus, understanding the motivations and decision-making process of medical tourists is key to directly targeting their behaviour. UK outbound medical tourists who return with complications mostly travelled to Turkey [6, 27], travelled for cosmetic or bariatric procedures [27], and are women [6, 20, 27] aged between 22 and 59 years (with a mean age of 35–36) [6, 20]. Existing evidence indicates that cosmetic and bariatric medical tourists are *pushed* away from receiving care from their domestic country due to factors such as recommendations from social networks, limited access and high cost of private care, and *pulled* toward specific destination countries due to lower cost, higher perceived expertise, and shorter waiting times [28]. Furthermore, medical tourists have been reported to conduct extensive, mainly online, research before arranging surgery [29]. However, there is a need to synthesise the available literature to gain an integrative understanding of the decision making of medical tourists, from contemplation to planning of and receipt of surgery. Existing reviews primarily adopt an industry perspective and seek to promote MT rather than to reduce the harms of MT [30, 31], lack systematic methods [28], or reduce findings to motivations [7, 17, 28, 32]. An existing integrative systematic review did not exclude studies based on type of MT or outbound country, or compare findings based on these two dimensions [29]. Thus, it is uncertain how findings may differ for medical tourists travelling from countries with universal healthcare, specifically for both high-risk and common MT procedures (bariatric and cosmetic tourism) that have limited or no universal healthcare coverage. The aims of the current systematic review were therefore to synthesise the current literature around the decision-making process of bariatric and cosmetic tourists travelling from countries with universal healthcare.

## Methods

Reporting of this systematic review adheres to PRISMA guidelines [33], including the interim reporting guidance for rapid reviews. Due to the policy relevance of the review, a rapid review approach was adopted, adhering to Cochrane guidelines for rapid reviews [34, 35]. Specifically, this rapid review deviated from standard Cochrane methodology through limiting included studies to English language, only carrying out a proportion of independent screening (dependent on good inter-rater reliability), and performing single data extraction with checks by another author. Policy partners from the Department of Health and Social Care were consulted after the authors drafted a protocol a priori, and the focus of cosmetic and bariatric tourism was jointly agreed to ensure policy relevance. Prior to screening, the protocol for the current review was pre-registered via the International Prospective Register of Systematic Reviews (PROSPERO registration number: CRD420251000992). In accordance with PROSPERO guidelines, PROSPERO was searched for similar registrations which confirmed that there were no protocols with a similar scope. The protocol was amended after full text screening. Details of the amendment are provided under ‘Deviations from the registered protocol’.

### Eligibility criteria

The SPIDER (Sample, Phenomenon of Interest, Design, Evaluation, Research type) framework [36] was applied to frame the inclusion and exclusion criteria (see Supplementary Material 2). Studies were included if they sampled individuals from countries with universal healthcare who had either engaged in or considered engaging in MT for invasive procedures with little or no universal healthcare coverage in their home country (Sample). Universal healthcare was defined as affordable access to quality healthcare for all, when and where they need them [37], with countries checked using the World Population Review tool: https://worldpopulationreview.com/country-rankings/countries-with-universal-healthcare. Thus, the focus of this review was MT specifically related to cosmetic (including dental work) and bariatric procedures (Phenomenon of Interest). Studies which focused on patients sent abroad through cross-border care arrangements were excluded (Phenomenon of Interest). Primary studies that employed qualitative, quantitative, or mixed methods were included (Design). Systematic reviews and other evidence syntheses were excluded from analysis, although their reference lists were screened for eligible primary studies (Design). Studies were included if they assessed the motivations, experiences, and factors influencing patients’ decision-making around MT specifically for bariatric and cosmetic tourism, and studies that merely measured the prevalence of MT were excluded (evaluation). For all criteria, studies were included if > 50% of participants were eligible, or if the data could be extracted for eligible participants only. For example, if a study sampled mostly cosmetic tourists from the UK although some were from the USA, the study was included. Finally, both peer reviewed studies and grey literature were included, with no limitations on year of publication (Research Type).

### Search strategy

Three databases (MEDLINE^®^ (via OVID), PsycINFO (via OVID), and Web of Science (via Clarivate)) were searched on the 24th February 2025. Prior to the searches, scoping searches were completed to develop the search terms, and guidance was sought from an information specialist within the Library Liaison team at Newcastle University on the relevance of information sources and search terms. The resulting search strategy (see Table 1) was based on Phenomenon of Interest and Evaluation and was applied to title and abstract (see Supplementary Material 3 for the specific search applied to each database). There were no restrictions based on publication date, although due to a lack of available resource for translation services, the search was limited to articles published in English language. Additionally, grey literature was included due to the policy relevance of the topic, through applying the search strategy to Overton (searched on the 26th February 2025) and hand searching google scholar. Additionally, forwards and backwards citation searching was applied to ensure a comprehensive search.

**Table 1:** Search strategy. Columns are separated with the Boolean operator ‘AND’. The full search strategy for each database is supplied in Supplementary Material 3

Medical tourism (phenomenon of interest)	Decision making (evaluation)
“Medical tourism” OR “Hospital outshopping” OR “Health tourism” OR “Cosmetic tourism” OR “Aesthetic tourism” OR “Overseas treatment” OR “Treatment overseas”	Decision* OR Factors OR Attitude* OR Perspective* OR Motivat* OR Choice OR Ch$ose OR Concept* OR experience* OR attitude* OR perception* OR belief* OR opinion* OR view* OR perspective* OR voice* OR value* OR Story OR Stories OR Storytelling OR choice* OR account* OR reason* OR themes OR thematic OR narration OR “exploratory research” OR Theor* OR Intention* OR “accounts” OR “an account” OR Ethnograph* OR Autoethnography OR Benefit*
OR Abroad OR Outsourcing OR Touris* OR Transnational OR Overseas OR “Cross$border” AND Dental OR dentist* OR bariatric OR “gastric sleeve” OR “vertical sleeve gastrectom*” OR “gastrectom* sleeve*” OR “sleeve gastrectom*” OR “gastric bypass” OR “stomach bypass” or “roux-en-y” OR “RYGB” OR “biliopancreatic bypass” OR “duodenal switch*” OR “duodenum switch*” OR “biliopancreatic diver*” OR “gastric restrict*” OR “gastric band*” OR lap$band* OR “laparoscop* band*” OR “gastric balloon” OR “brow lift” OR Face$lift OR “Neck lift” OR “breast augmentation” OR “Breast implants” OR “Breast lift” OR “Brazilian Butt Lift” OR liposuction OR “patient mobility” OR “Rhinoplasty” OR “Rhytidoplasty” OR “Genioplasty” OR “otoplasty” OR “Blepharoplasty” OR “abdominoplasty” OR “tummy tuck” OR “Plastic surgery” OR lipoinjection OR “Buttock* augmentation” OR “Buttock* enhancement” OR “Gluteal fat grafting” OR “Buttock* implants” OR “Esthetic surgery” OR “Aesthetic surgery” OR “Body contouring surgery”	

### Study selection

Search results were exported via a RIS file and uploaded to Endnote for removal of duplicates, before being uploaded to Covidence for screening (conducted by BN). As noted in Cochrane guidelines for rapid reviews, dual screening of a proportion of articles is sufficient provided that inter rater reliability is good [34]. An independent reviewer (LT) dual screened 10% of the screening based on title and abstract. Using Cohen’s kappa statistic [38] and using the conservative parameters from Altman [39] to assess inter-rater reliability, inter rater reliability for title and abstract was judged to be good (*k* = 0.78, SE = 0.081, 95% CI: 0.620–0.936) and all remaining articles were single screened by one reviewer (BN). Again, 10% of articles were screened based on full text (by either LM or DR). The kappa statistic for full text screening was not available as all dual screened articles were excluded, however agreement with the second reviewer was 100% and 95% respectively, which was judged to be sufficient. Any discrepancies were resolved through discussion between reviewers until a consensus was reached, and where necessary, consultation with a third reviewer. Independent screening and resolution of discrepancies was conducted and logged via Covidence.

### Data extraction and quality appraisal

The bespoke data extraction form (see Supplementary Material 4) was developed in accordance with the specific aims of the current review, using guidance from Cochrane [40, 41]. Only data relevant to the aims of the review were extracted. In accordance with Cochrane Guidance for rapid reviews [34], one review members (BN, LT, LM) extracted the data from each study and the extracted results of included studies were reviewed and verified for accuracy and completeness by a second reviewer (DR, LM, BN). The data completed extraction form was uploaded to OSF, in accordance with open science principles.

Due to the large range of potential study types included in the systematic review, the Mixed Methods Appraisal Tool (MMAT) was employed to assess the quality of included qualitative, quantitative, and mixed methods studies [42]. The tool separates included studies into five categories and different items are rated depending on the study type (see Supplementary Material 5). In accordance with guidance for applying the MMAT [42], overall quality scores were not calculated for each included study, and instead the main potential sources of poor quality and bias within each study are acknowledged. To ensure a comprehensive review, no studies were excluded based on quality. Quality appraisal was conducted by one reviewer (BN) and reviewed and verified by a second reviewer (LT), with any disagreements resolved through discussion.

### Patient and public involvement and engagement

Experts by experience (*n* = 4), namely individuals who had engaged in or considered engaging in cosmetic or bariatric tourism, were consulted throughout the project to ensure its relevance and validity. Two experts by experience had received cosmetic surgery abroad, one had considered bariatric surgery abroad, and another had received bariatric surgery abroad. One individual was consulted prior to the searches to advise on the review aims and scope. Four individuals (including the same previously consulted individual) met with the primary researcher (BN) after data analysis to corroborate the findings and ensure accurate and relevant interpretation. For example, the experts by experience noted that medical tourists often cannot afford to pursue private surgery within their home country, and MT enables them to receive their desired surgery. Subsequently, the interpretation of the review findings was amended to recognise that a harm reduction approach may be most realistic. Additionally, the experts by experience advised on directions for future research.

### Deviations from the registered protocol

Originally, any studies that did not adhere to the inclusion criteria were only going to be included if they specifically included people who met the inclusion criteria, or stratified data for these participants. After full text screening, to optimise the useful data included in the review, the inclusion criteria were broadened to include studies where most participants were eligible in terms of country of origin and type of surgery sought. Studies were also included if a minority of participants were eligible for the review and their data could be extracted. Thus, all studies were re-screened at full-text stage against the updated inclusion criteria. In this case, only the data relating to eligible participants was extracted.

### Data synthesis

Cochrane guidance recommends Thematic Synthesis to synthesise qualitative data for reviews for use in decision-making which integrate quantitative data [40]. Thus, the findings sections of each included study (including quotations) were imported as a separate Word case file into Nvivo for Thematic Synthesis [43] conducted by one researcher (BN). Firstly, inductive line by line coding was initially applied to the whole dataset, identifying codes that were either freely coded assigned to a coding ‘tree’ or hierarchical structure of codes. These ‘trees’ were both developed throughout coding and after coding to generate descriptive themes. These were then further integrated and developed, informed by notes taken throughout the analysis process, to produce analytical themes. Analytical themes were further refined through discussions with the core review team. Additionally, where possible, findings were compared by type of MT (cosmetic, bariatric, or mixed sample) and country of residence of medical tourists.

Heterogeneity in the included inferential studies, according to the theories, scales, and factors tested, meant that meta-analysis was not possible. Instead, narrative synthesis was applied, following the SwiM guidance [44]. Since the study was exploratory, the strategy for data synthesis of quantitative studies was planned after data extraction, informed by the available literature. Thus, studies were grouped by design (descriptive or inferential), type of MT (cosmetic, bariatric, or mixed) and whether the data collection concerned intentions or retrospective behaviour. The standardised metric was chosen to be a p value, given the complexity of included inferential study designs.

Triangulation of both data types occurred through ‘*qualitising*’ the quantitative data [45]. Specifically, due to its scant availability, quantitative data was mainly used to supplement and validate the qualitative findings. To triangulate the findings of both data types systematically, qualitative and quantitative findings were mapped against each other using the matrix method [46]. Namely, statements about how decision making around MT occurs were generated from the findings of the qualitative research and plotted against either the number of quantitative studies that support each statement (in the case of motivations), or the available supporting or conflicting quantitative evidence. Due to the heterogeneity of the include quantitative data, the results of the plotting exercise are mainly described narratively under the ‘Triangulation’ section of the results.

To avoid repetition within the results section, findings from the included quantitative studies are only discussed in relation to the qualitative findings (triangulation). However, the full narrative synthesis in accordance with the SwiM guidance and the data that informed the narrative synthesis are available in the Supplementary Material. Thus, the results section first presents the thematic synthesis of included qualitative studies, followed by triangulation of findings. Where differences were observed, comparisons between types of MT (cosmetic and bariatric) and differences between countries are noted throughout.

## Results

### Search results

**Fig. 1:**
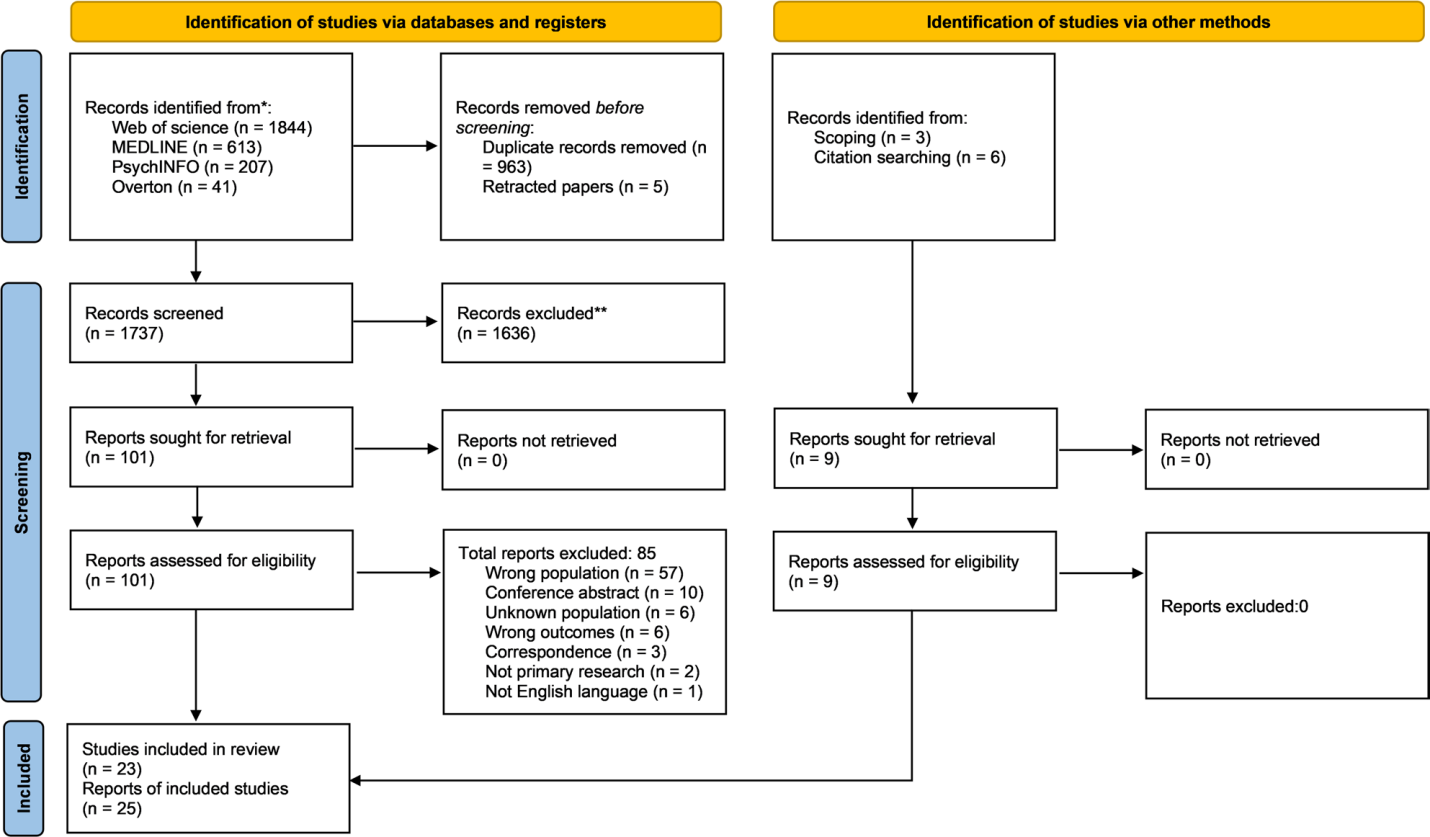
PRISMA diagram depicting the review search and screening process

The PRISMA [33] diagram in Fig. 1 displays the search and screening process. The search identified 2705 results. Upon screening based on full text, articles were most often excluded based on the type of MT or the country of origin of medical tourists (see Supplementary Material 6 for a full reference list of studies excluded based on full text and the reason for exclusion). 25 articles were included in the current review (representing 23 distinct samples).

### Characteristics of included studies

The main characteristics and key findings of each included study are displayed in Supplementary Material 7. Included studies were most commonly qualitative (*n* = 12) [47–58] or quantitative (*n* = 11) [19, 59–68], with only two mixed methods studies [69, 70]. Studies were published between and 2010 [67] and 2025 [59, 68] and represented a total of 3408 participants; made up of 311 qualitative and 3097 quantitative participants (not including participants that data was not extracted for within three qualitative studies [48, 49, 54], unknown numbers of participants that took part in focus group or creative methods [49, 50], and accounting for two studies that utilised existing samples included in the review [52, 58]). Most often, participants were sampled from their home country [19, 48, 49, 56, 57, 60, 61, 63, 65–69] although many studies conducted sampling within the destination country [54, 59, 62, 64, 70]. Studies mostly focused on cosmetic tourism [47, 54, 56–58, 60–68], with a minority focusing on a broader range of MT [48–50, 53, 55, 69, 70] or bariatric tourism [19, 51, 52, 59]. Overall, the home countries of medical tourists sampled in the included studies were mainly mixed [47, 50, 53–55, 58, 59, 64, 70], followed by the UK [49, 60, 65, 67, 68], Canada [19, 51, 52, 57], and China [63, 66, 69], with one study each from Italy [61], Australia [56], and Japan [62]. A minority of included studies focused specifically on medical tourists who had experienced complications [57, 65, 68].

Qualitative data from 14 included studies were included, which presented data from 12 samples, with sample sizes ranging from 8 [56] to 103 [50]. Most studies utilised semi-structured interviews [47–52, 54, 56–58, 69, 70], although some combined this data collection method with focus groups [49] or creative methods such as photovoice [50, 58], or utilised publicly available online narratives collected from blog posts and forums [53, 55]. Most utilised thematic analysis [48–52, 54, 56–58], although other approaches to analysis included content analysis [53], narrative analysis [55], applying software to detect patterns in the text [47], and the laddering technique [69].

13 sets of eligible quantitative data were extracted. Sample sizes ranged from 6 [65] to 1047 [66]. Of these, only two focused on bariatric tourism [19, 59], whilst most focused exclusively on cosmetic tourism [60–64]. All quantitative data was cross-sectional collected through surveys, and most merely reported descriptive data of relevance [19, 59, 61, 65, 67, 68]. A minority conducted inferential analyses [60, 62–64, 66, 70], which mainly consisted of a combination of confirmatory and/or exploratory factor analysis [60, 62, 63, 66], and structural equation modelling [62–64, 70]. Most frequently, postoperative medical tourists were asked to recall their experiences retrospectively [19, 57, 59, 61, 65, 68, 70] (with some specifically focusing on individuals who had experienced complications [57, 65, 68]), or prospective medical tourists were asked to rate their intention in engaging in MT in the future [60, 62–64, 66, 67] (with some specifically sampling participants who were already intending on engaging in MT [63, 64, 66].

### Quality of included studies

See Supplementary Material 5 for the quality appraisal scores for each included study. Qualitative studies generally outlined a clear research question, and all adopted a suitable approach to answer it, although some lacked clarity about the data collection methods by not providing or explaining the topic guide. Qualitative studies were inconsistent in the quality of data analysis and interpretation, and often did not support findings with sufficient data, provide sufficient interpretation of the data, or explain the process of data analysis in sufficient detail. Only one study answered ‘Yes’ to all quality appraisal items [56].

Quantitative studies all provided a clear research question, and most authors conducted appropriate statistical analysis to answer it. However, many studies did not provide sufficient information to judge whether the risk of nonresponse bias was low, or due to a lack of provision of the survey items, whether collected data was sufficient to address the research question. Many of the included quantitative studies presented with concerns around representativeness of the sample, validity of the survey items, and specific recruitment sources and their appropriateness to answer the research question. For example, many of the included studies assessed motivations for MT and either did not provide details of the survey or reported extremely brief surveys with minimal response items and no piloting or testing for reliability and validity.

The two included mixed methods studies [69, 70] indicated many sources of concern for study quality and risk of bias, across all quantitative data collection, qualitative data collection, and triangulation. Whilst both studies provided a clear research question and collected data to address it including an appropriate statistical analysis, concerns included insufficient rationale for employing a mixed methods design, insufficient information about the representativeness of the quantitative data, and minimal triangulation of data sources.

### Findings from qualitative studies

A summary of the themes, sub-themes, and supporting data is provided in Table 2.

**Table 2:** Summary of qualitative themes, sub-themes, and supporting data resulting from the Thematic Synthesis

Theme	Sub-themes	Key codes	Key quotes
Medical Tourists as Constrained Consumers: Decision-Making under Systemic Healthcare Limitations	• Circumventing systemic healthcare limitations in home country • Sense of urgency for treatment • Focused on cost, expertise, and value for money • The specific decision-making pathway of medical tourists is unclear • Choosing a specific destination (post choice to engage in medical tourism)	• Primary motivator is availability in home country • Chooses abroad to receive the treatment faster • Cost as the primary motivator • Perceived higher quality and advancement of care abroad • Considered surgery for a long time • Initial consultation perceived as an opportunity to build trust or not • Cheap cost of travel and treatment as the primary motivator for country choice	“[private] Surgeons in home countries were characterised as aloof, inattentive, uncaring and as seeing patients as ‘*walking cheque books’*.” (Holliday, 2014, Cosmetic surgery) “*They [doctor] said your BMI’s not high enough. Your BMI has to be at least 35 and I think I was 32 or something. So they said nope*,* you’re not high enough. And then my weight kept going up and I said ‘oh great*,* this is nice and now I have to see my weight keep going up’. But it wasn’t 35*,* it was 33 or 34 so it was close to 35 and they couldn’t refer me and I couldn’t do anything because my BMI wasn’t high enough* (Participant #17). Another participant felt generally frustrated over not being able to access care because of her weight: “*okay you’re not going to help me because I’m not fat enough?! That doesn’t make any sense*.” (Participant #6)” (Jackson, 2018) “Cost was discussed by all of our respondents and in almost all cases was an important motivator for travel abroad… Cost played a particularly strong role amongst cosmetic tourists and the dental tourists interviewed where these had no cultural or familial ties to the destination to which they travelled… While cost was important to our bariatric respondents, it did not take precedence to the same extent, rather being a contributing factor, albeit an important one, alongside others.” (Hanefield, 2015)
Crowdsourcing Trust: Peer-Produced Evidence and Trusting strangers in the absence of support	• Medical tourism as a lonely and self-navigated process • Support from friends, family, and domestic healthcare professionals is highly valued but rare • Lack of support creates a market for facilitators • The internet as a help and a hindrance • Anecdotal evidence as the most powerful • Use of social media and forums primarily after intending to engage in MT, for social support and choosing a surgeon	• Medical tourists may not tell family, friends, or domestic healthcare professionals about decision to travel abroad due to stigma and fear of judgement • Strong influence of cultural or familial ties on the chosen destination country (diasporic MT) • Many medical tourists utilise a facilitator to coordinate their MT experience • Internet as a source of information Cost of the surgery abroad could be unclear • Medical tourists start their MT journey online with websites High value placed on hearing from previous patients’ experiences	“The idea around ‘making decisions independently’ was consistent across our participants. In fact, limiting disclosure of procedures was a common feature across our participants’ accounts due to their expectations of a negative response.” (Prasad, 2024) “While websites played an important role in research, many of our interviewees also emphasised the importance of contact with peers and previous patients via social media. Most participate in Facebook, forums or YouTube, or at least ‘lurk’ on them. We note that cosmetic surgery tourists are generally not content with the singular authority figure of the surgeon, or the advertising of hospitals and clinics, and to augment these modes of information they rely on each other” (Jones, 2014) “*I used what this woman had told me [online]. She told me the clinic she went to*,* the doctor*,* and then I went and researched from there. But I totally relied on this woman*.” (Jackson, 2019)
Informed but Invested: Navigating Risk Through Emotional Commitment	• Medical tourists believe they are aware of the risks • Emotional investment- strong motivations for surgery • Actions to minimise risks • Reducing cognitive dissonance- medical tourists form beliefs to minimise perceived risks	• Aware of the possible risks when decision making • Hygiene is greatly important • Concerns about language barrier but did not prevent MT • Wide variety of reasons for wanting surgery- mainly centre around wanting to look/ be ‘normal’ • Medical tourists undertake extensive research • Once a decision has been made, information is only affirming • Optimism that negative outcome won’t happen to me	“Another patient told us of following, via Facebook, a group of cosmetic surgery tourists’ surgery and recovery in the weeks leading up to her own trip: *‘It’s been really helpful. Some have been having a bad time*,* they’ve been in more pain than they expected*,* whereas some of them are out on the back of elephants*,* so it’s good to see how everybody is different but it’s not all… and one girl got*,* like*,* an infection*,* you know. So it’s not showing only the good side of the story. I hope none of this happens [to me]. I’m very excited’* (Sue, Australia to Thailand). For Sue, seeing people miserable with infections alongside those well enough to take post-surgical elephant rides gave her confidence. While she hoped she would have a good experience, she was able to moderate that against the possibility that she might not.” (Jones, 2014, Cosmetic surgery)

### Theme 1: Medical tourists as constrained consumers: decision-making under systemic healthcare limitations

#### Circumventing systemic healthcare limitations in home country

Medical tourists initially sought treatment abroad after they experienced or assumed ineligibility for universal healthcare in their home country [47, 49, 51, 52], thus ‘*circumventing*’ the system [51]. Cosmetic tourists mostly assumed ineligibility and had not initially sought universal healthcare before considering treatment abroad [49]. By contrast, for bariatric tourists, their desire to travel abroad for weight loss surgery was most often to circumvent the strict eligibility criteria for access to universal care in their home country, the knowledge of which was either gained through word of mouth, consultation with HCPs in cosmetic tourists’ home country, or personal assumptions [49, 51]. Subsequently, bariatric tourists noted how they were often stuck between struggling with their weight yet did not meet the BMI requirement for weight loss surgery within the universal healthcare system [51]. In other cases, the specific procedure that best suited a bariatric tourist’s needs and preferences was not available locally [49, 51]. Subsequently, individuals felt a lack of control of their body, health, and care pathway, and mistrust and dissatisfaction with universal healthcare [49, 51]. Thus, exploring treatment abroad allowed access to bariatric treatment that was either limited or unavailable within the universal healthcare system. For all types of MT, a minority of studies discussed a dissatisfaction with the quality of customer service of domestic private healthcare [48–50] (often linked to those with cultural and familiar ties elsewhere [49]) or a lack of availability of the desired procedure [47, 49], leading them to seek treatment abroad. One study identified that many cosmetic tourists were motivated by the availability of postoperative care, indicating a perceived lack of postoperative care in one’s own country [57].

#### Sense of urgency for treatment (bariatric only)

Also, many bariatric tourists noted a lengthy waiting list and pre-operative eligibility process and felt a sense of urgency for the sake of their health and everyday functioning, thus exploring treatment abroad allowed for timely treatment but also an opportunity to reclaim control over one’s own health [51, 52]. This urgency meant bariatric tourists were willing to forgo postoperative care in their home country [52]. The same urgency to act now to avoid negative health consequences was not observed within the qualitative data for cosmetic tourists.

#### Focus on cost, expertise, and value for money

Given the pivot to private treatment, individuals became consumers instead of passive patients, considering their preferences, needs, and limitations to choose a surgeon. Medical tourists primarily discussed reduced cost as their primary motivator for pursuing treatment abroad instead of privately in their home country [47–49, 53–55, 57], enabling treatment that likely was not affordable to them otherwise. However, many medical tourists noted perceived expertise and experience of surgeons and quality of care abroad as a large influence of their choice to receive treatment abroad [47, 49–51, 54, 56, 58, 70], with some noting expertise as a more prominent motivator than cost. Many medical tourists noted that surgeons abroad were pioneering and advanced compared to domestic surgeons, offering the latest technologies and procedures [51, 70]. For some medical tourists, surgery abroad was perceived to provide improved customer service, facilities, and hygiene [49, 57, 69]. For many, this perception was likely heightened by lower cost which indicated better value for money [47, 49], which for some bariatric tourists was the most appealing motivator for treatment abroad [49]. Indeed, cost appeared to be one of multiple motivators for bariatric tourism rather than the primary motivator, unlike for many cosmetic tourists who were often mainly motivated by cost [49].

#### The specific decision-making pathway of medical tourists is unclear

The studies lacked detail on whether medical tourists select private healthcare as a preferred option prior to seeking treatment abroad, whether medical tourists select a destination country before selecting a provider, and at what point MT sought information to inform their decision-making.

#### Choosing a specific destination (post choice to engage in medical tourism)

Some medical tourists appeared to decide to go abroad for treatment before selecting a specific destination. In these cases, factors that influenced destination choice included cost (including both treatment and staying in the country), distance and accessibility from home country, expertise, familiarity, cultural and familial links to the destination country, communication and responsiveness, availability of facilitators, and availability of postoperative care [49, 50, 53, 55, 69]. For bariatric tourists, proximity to the destination country and travel routes was particularly important, as they considered potential risks of postoperative travel. A minority of cosmetic tourists noted tourist attractions and opportunity for vacation as a factor in their decision making [49, 50, 53, 69], which may be interpreted as a way of reasserting agency over the MT decision-making process that was otherwise shaped by the inaccessibility of universal healthcare and the unaffordability of private domestic treatment.

### Theme 2: Crowdsourcing trust: peer-produced evidence and trusting strangers in the absence of support

#### Medical tourism as a lonely and self-navigated process

MT means independently seeking treatment outside of the support of individuals’ own healthcare system. Due to anticipated or experienced judgement from domestic healthcare providers, medical tourists often do not inform or consult with them before engaging in MT [52]. Of those who did, medical tourists often discussed experiences of judgement that prevented them from further engaging with domestic HCPs throughout their MT journey [52, 56]. Similarly, medical tourists experienced or assumed judgement from their family and friends around their decision to engage in MT, and subsequently only shared their decision to engage in MT with a limited selection of friends and family or chose to disclose their decision only after engaging in MT [52, 56, 70]. Medical tourists that did discuss with friends or family often reported negative reactions [52]. Stigma around MT appeared to occur on a continuum; whilst MT for dental procedures carried little experience of stigma [47], bariatric tourism was particularly stigmatised due to additional judgement around weight and weight loss efforts [52]. At its worst, fear of stigma which led to isolated decision making meant that medical tourists received inadequate follow-up care [52]. Subsequently, the MT journey is a lonely and self-navigated process.

#### Support from friends, family, and domestic healthcare professionals is highly valued but rare

Nonetheless, medical tourists appreciated social support from friends and family, including as a companion to treatment [54]. Whilst only one study reported that some medical tourists received support from a domestic HCP, this support meant that medical tourists were able to access postoperative care domestically and contingency plan in the event of complications [52].

#### Lack of support creates a market for facilitators

Many medical tourists are unfamiliar with their destination country and often face challenges navigating a foreign environment and language [50]. Thus, medical tourists often utilised facilitators who occupy a paid sales role and often live in the destination country [49, 55], acting as ‘*brokers*’ between medical tourists and providers [50]. Facilitators are often previous medical tourists and provided support guidance on expectations and the medial tourist experience [49, 50, 53]. Indeed, facilitators appeared to be particularly helpful when support from other sources was lacking (excluding diasporic tourists [53]), as medical tourists frequently told positive accounts of their interactions with them [55].

#### The internet as a help and a hindrance

Additionally, medical tourists conducted their own research online to inform their decisions, particularly for those who did not receive recommendations from friends or family about a specific MT destination or surgeon [56]. Medical tourists accessed provider websites to conduct their own extensive research, comparing factors such as cost, qualifications, and services [55, 56, 58]. However, medical tourists noted uncertainty around provider websites and craved social support and the need to see *‘physical results’* (proof of positive outcomes from previous patients) [56]. Medical tourists subsequently utilised social media, forums, and other online groups to interact with other medical tourists, both for social support and for practical information [55, 56, 58]. However, medical tourists noted that providers could infiltrate such social networks with advertisements and promotions which were often covert [49, 50].

#### Anecdotal evidence as the most powerful

Whether online or through recommendation from friends and family, anecdotal evidence was most valued by medical tourists [47, 49, 50, 52, 53, 56, 57]. Online anecdotal evidence included testimonials, before and after photos, reviews, and recommendations from influencers [47], and medical tourists were keen to view every step of the MT process online through the perspective of a MT [47, 58]. Expertise and quality was judged through stories and feedback from previous medical tourists, to a greater extent than from formal qualifications [49, 50]. Indeed, many medical tourists selected their provider based on recommendation from a peer online [49, 52, 56]. This some evidence that providers exploited the value of anecdotal evidence for medical tourists, by facilitating contact with previous patients [49].

#### Use of social media and forums primarily after intending to engage in MT, for social support and choosing a surgeon

Much of the use of social media and forums described by medical tourists appeared to affirm rather than dictate individuals’ decision to engage in MT [56]. Medical tourists accessed social media and forums to decide on a specific provider and surgery, although it appears that medical tourists already intend to engage in MT before social network use. Examples of post-intention use included use of social networks to augment information from providers, to choose a surgeon or destination country, and for reassurance and emotional support [49, 50, 52, 55, 56, 58]. Additionally, one included study also suggested that rather than merely as vectors for social interaction and sharing of information, use of social media and forums helped shape medical tourists’ identities [58], leading to continued use after receipt of surgery that continues the cycle of previous medical tourists sharing their experiences online.

One exception from this theme was diasporic medical tourists, who relied on their existing networks and knowledge of the destination country to arrange their journey.

### Theme 3: Informed but invested: navigating risk through emotional commitment

#### Medical tourists believe they are aware of the risks

As discussed, MT is a self-navigated process within which the medical tourist assumes responsibility for their care. As such, medical tourists tend to demonstrate at least some awareness of the potential risks to be mindful when selecting treatment [47, 69, 70], including complications of the surgery [70], malpractice [69], and concerns about hygiene and quality of care [54, 57, 69]. Many medical tourists feared ‘*botched*’ surgery even after selecting a surgeon [70]. medical tourists were aware of predatory behaviour of surgeons on social media and forums and of unreliable and untrustworthy information often presented on treatment providers’ websites [56]. Risks less frequently discussed were the specific risks associated with receiving treatment abroad, such as navigation of the legal system [47], navigating rehabilitation and aftercare [70], a language barrier [47, 70], and travelling after surgery [70].

However, MT perceptions of safety and risk were often not based on critical appraisal of evidence, and more often were formed through assumptions of word of mouth [49]. Also, medical tourists noted a lack of transparency of providers around the risks of surgery, making informed decision making difficult [57]. There was also evidence of medical tourists acting recklessly such as not checking government guidance [56] or adhering to postoperative care advice [50], perhaps indicating low risk perception.

#### Emotional investment- strong motivations for surgery

Despite this, medical tourists were strongly motivated to receive treatment which led to them managing the risks instead of avoiding engaging in MT. Particularly for cosmetic tourism, medical tourists possessed a strong desire to look ‘*normal*’ [50] and surgery was closely tied to self-identity and sense of self [56], which may have led to some disregard of the potential risks of engaging in MT. Subsequently, awareness of some of the risks of MT did not appear to be sufficient to deter medical tourists.

#### Actions to minimise risks

To cope with the perception of risk, medical tourists most often engage in extensive research [47, 50, 53, 55, 56, 58], which reassured them of their decision [47, 58]. Acknowledging that online information about surgeons can be untrustworthy, medical tourists relied on anecdotal evidence (as above). Additionally, medical tourists made choices that decreased their perceived risk, such as choosing familiar destination countries [70], organising surgery as quickly as possible to avoid time spent considering risks [50], or visiting the surgeon for an in-person consultation [56], which decreased perceived risk by building trust (even if misguided).

#### Reducing cognitive dissonance- medical tourists form beliefs to minimise perceived risks

However, medical tourists - particularly those who had already decided to travel abroad - sometimes ignored guidance and information about risk and only attended to information that affirmed their decision. To illustrate, some medical tourists described instances of viewing evidence that the procedure was not safe [58] or feeling dissatisfied with the safety of the surgeon after consultation [56], yet still deciding to go ahead with the procedure [56, 58]. This was explicable by the beliefs medical tourists formed to ease the psychological discomfort of information that challenged their emotional investment of receiving surgery. For example, medical tourists believed that complications wouldn’t happen to them [58], that the risks were the same no matter the country [70], or focused on supportive information of their decision such as the impressive facilities and service [56]. These beliefs helped medical tourists to avoid cognitive dissonance by dampening their risk perception. Additional supportive beliefs included that medical tourists were benefitting the health care providers and wider healthcare system in the destination country [47].

#### Triangulation

The extracted quantitative data that supported the narrative synthesis and the narrative synthesis in accordance with the SwiM guidance is shown in Supplementary Material 8 and 9, respectively. Triangulation of qualitative and quantitative studies are organised according to the themes and sub-themes generated from the thematic synthesis, where relevant data is available.

### Theme 1: Medical tourists as constrained consumers: decision-making under systemic healthcare limitations

#### Circumventing systematic healthcare limitations in home country

Table 3 summarises the available descriptive data relating to motivations. There was no quantitative data relating to differences between bariatric and cosmetic tourists, including whether medical tourists seek universal healthcare before considering abroad. Long waiting times was a motivator exclusive to bariatric tourists [19, 59] (see Table 3), although actual or assumed ineligibility [19, 59] was also discussed by one study of cosmetic tourists [65]. The measures of perceived behavioural control within the conduced quantitative studies [62, 63] did not directly address the assertion by the qualitative data that engaging in MT allows individuals to exert control over their treatment.

**Table 3:** Matrix of motivations for MT identified within the qualitative research mapped against motivations identified within quantitative descriptive studies

Proposed motivation	Supporting evidence from quantitative research (number, type of surgery)		Conflicting evidence from quantitative research	
	No. studies	Surgery type	No. studies	Surgery type
Lower cost	5	T	No references	
Availability of postoperative care	1	C	No references	
Long waiting times	1	B	1	B
Recommendation	1	C	3	T
Customer service	1	C	1	B
Quality of care	No references		1	C
Shorter rehabilitation period	No references		1	C
Ineligibility (actual or assumed)	No references		2	B

Key: *≥* 70% of participants selecting that information source/ motivation = supporting evidence, < 70% = conflicting evidence. No references = no studies that measured and reported frequency according to proportion. For surgery type: ‘C = cosmetic only, B = bariatric only, T = triangulated across surgery types’

#### Focused on cost, expertise, and value for money

Lower cost abroad was frequently identified as the most common reason for MT within descriptive studies (or one of the most common reasons [65]), across both bariatric [59] and cosmetic [57, 61, 67, 68] MT. Indeed, the risk perceptions of prospective cosmetic tourists around MT were the highest for risks relating to cost, compared to risks relating to complications, poor quality surgery, or travelling abroad [66]. There were no quantitative data that explicitly reported perceived expertise as a motivator, although quantitative studies reported related motivations. For example, perception of greater quality of postoperative care abroad was the most common motivator for MT after cost for cosmetic tourists [57]. Customer service was cited by two studies as a motivator [57, 59], although was much more highly reported in cosmetic tourists (73%) [57] compared to bariatric tourists (24%) [59]. Perceived quality of care was also cited by 45% of cosmetic tourists as a reason for MT [57], which subsequently predicted likelihood of revisiting a surgeon in another study [70]. An additional but less frequent concept to cost alone was value for money, as some medical tourists felt that the costs of surgery domestically was unjustified [61]. Other motivations relating MT as a service were frequently cited but rarely selected by 70% or more participants (see Table 3).

Additionally, the qualitative data suggested that opportunity for vacation or tourism appears to be secondary or complementary to core factors such as cost, which was largely supported by the quantitative data. Although one study reported that nearly half (49%) of respondents were equally motivated by both cosmetic surgery and vacation purposes, vacation-related risks, such as insufficient opportunity for leisure, were rated as less concerning than cost-related risks [66]. However, in another study, almost one fifth (18.1%) of respondents indicated interest in MT due to the opportunity for a vacation, although the sample was within the general population so was likely not representative of those considering MT [67]. No quantitative studies on bariatric tourism reported vacation as a motivator.

Further, the qualitative data suggested that whilst cost is the primary motivator for most cosmetic tourists, the relative importance is lower for bariatric tourism and just one of multiple motivations to engage in MT. The included quantitative evidence was inconclusive; whilst one study of bariatric tourists identified eligibility and availability as the most common reasons for MT [19], cost was still the most common reason for MT in another [59].

#### The specific decision-making pathway of medical tourists is unclear

The materials used to apply the Theory of Planned Behaviour (TPB) within the scant inferential quantitative studies [62, 63] focused on MT (e.g. ‘*Medical facilities abroad are better than in my own country’* [63]). Therefore, the available quantitative studies are limited in their ability to discern whether medical tourists are focused on country (to receive surgery domestically or abroad) or specific surgeon (and country of the surgeon is an unintended consequence). Thus, the specific decision pathway of medical tourists remains unclear when triangulating the quantitative and qualitative data.

### Theme 2: Crowdsourcing trust: Peer-produced evidence and trusting strangers in the absence of support

#### Medical tourism as a lonely and self-navigated process

Whilst the decision-making journey was apparently lonely and self-navigated, both the qualitative and quantitative data suggested that medical tourists are often motivated to engage in MT through recommendation from a friend, which also often determines their specific choice of surgeon. The quantitative data demonstrated that many are also accompanied to their surgery by a family member or friend. Thus, the quantitative data illustrates a somewhat less lonely journey. This perhaps indicates two distinct decision-making pathways- one in which medical tourists possess a friend or a family member who is a medical tourist themselves and recommends MT to them and supports them through their decision-making journey, or another in which the medical tourist is alone in their journey and seeks affirmation and information online.

There was no quantitative data that addressed the finding from the qualitative data that bariatric tourists experience heightened stigma around MT due to additional judgements around weight.

#### Support from friends, family, and domestic healthcare professionals is highly valued but rare

Whilst the qualitative data indicated a common lack of support from family and friends, the quantitative data that applied the TPB identified subjective norms (which assessed opinions of friends and family) as a significant predictor of intention to engage in cosmetic tourism [62, 63], although one study found that subjective norms were the least influential compared to perceived behavioural control and attitudes [62].

#### Lack of support creates a market for facilitators

Although the qualitative data identified a prominent market for facilitators in the destination country of medical tourists, facilitators were not discussed in any of the included quantitative studies.

#### The internet as a help and a hindrance

The qualitative data situated decision making online, specifically starting with a broad focus including websites, with the reliance on social media and forums growing throughout MT’s decision-making journeys. Only descriptive quantitative data was available relating to the information seeking of cosmetic tourists, specifically. Whilst one study identified that 70% of potential medical tourists had accessed the internet when considering MT [67], another study identified more common sources including recommendation from friends or relatives (66.6%), pamphlets (55%) or communication with the provider’s office staff (55%) [61]. It is noteworthy that the available quantitative literature on information seeking was published between 2010 and 2012 [61, 67], perhaps explaining the lesser role of internet sources.

#### Anecdotal evidence as the most powerful

Receiving a recommendation was the second most selected reason for travelling abroad for cosmetic [61, 68] or bariatric [59] tourism after cost. However, unlike cost, only one study reported more than 70% to select it as a motivator [57] (see Table 3). As previously noted, subjective norms (measuring what friends and family would think of an individual engaging in MT) significantly predicted intentions to engage in MT [62, 63]. However, in direct contrast to travelling for health and wellness treatment, intention to engage in cosmetic tourisms was least influenced by subjective norms when compared to attitudes and perceived behavioural control, although all were significant predictors [62]. Also, a recommendation from a friend was the most selected reason for specific provider choice for cosmetic tourists [61]. Although the qualitative data indicated that medical tourists utilise a range of information sources but trust peers more than information from providers, there was no available quantitative data on the degree of trust MT’s have towards the different information sources that they use.

### Theme 3: Informed but invested: navigating risk through emotional commitment

#### Medical tourists believe they are aware of the risks

The qualitative data suggests that medical tourists consider engaging in MT for a considerable time period and attempt to make informed decisions by conducting extensive research (albeit largely informed by anecdotal evidence). The available quantitative data on the extent of MT’s pre-operative research is tenuous. 14% of prospective cosmetic tourists would not select their provider before departure [66], and most bariatric tourists had knowledge of bariatric of bariatric surgery tourism before their procedure [59].

The qualitative data indicates that although medical tourists are aware of the risks, the awareness does not prevent them from engaging in MT. On the contrary, quantitative data indicates perceived risk as a predictor of intentions to engage in cosmetic tourism, through the TPB constructs of attitudes, social norms, and perceived behavioural control [63, 70]. Also, perceived risk significantly predicted affective image of MT which in turn predicted image of the destination, and partially moderated the effect of satisfaction (with the care they received) on loyalty [70]. However, perceived benefit also significantly predicted all TPB constructs at the *p* < .001 level [63]. Also, another study of cosmetic tourists which segmented the sample using latent class modelling and the chi-square automatic interaction detection (CHAID) algorithm identified that most medical tourists perceive low levels of risk overall (39%) [66]. Only 22% were identified as ‘risk sensitive’, perceiving all four types of risk (related to cost, the medical procedure, the destination country, and travelling) [66]. Indeed, most prospective cosmetic tourists reported that they would stay in a hotel (63%) instead of a specialised accommodation for postoperative recovery, indicating a lack of risk perception relating to the surgery [66]. Of the risks that were discussed by participants within the included qualitative data, medical tourists appeared to be aware of the universal risks with surgery, rather than the specific risks associated with MT compared to receipt of domestic surgery. However, the segmentation of cosmetic tourists identified that 61% of the sample perceived some risks associated with MT, including specific risks associated with vacation such as immigration issues and complications after surgery [66]. Nonetheless, risks associated with unanticipated costs demonstrated the highest mean scores, affirming the qualitative finding that cost is prioritised over risk.

#### Emotional investment- strong motivations for surgery

No quantitative data assessed prospective MT’s motivation to engage in MT. A large proportion (39%) of the aforementioned segmented sample demonstrated low mean scores in all types of risk, and were the group who were willing to spend the highest amount of money on highly invasive cosmetic procedures such as a facelift [66].

#### Actions to minimise risks

No quantitative data addressed the behaviours that medical tourists engage in to reduce their perceived risk of MT, aside from one study which found that working with the provider to finalise medical tourists’ surgery partially mediated the effect of medical service quality on trust in receiving and intention to engage in cosmetic tourism [64].

#### Reducing cognitive dissonance- medical tourists form beliefs to minimise perceived risks

The qualitative data suggests that once individuals intend on engaging in MT, the way they process and cope with risk is affected through forming protective beliefs. There was no quantitative data that addressed altered beliefs to minimise risk perception. However, another descriptive study reported that most bariatric tourists (92.9%) believed their surgery had been successful, despite three medical tourists (23.1%) experiencing complications [19].

## Discussion

This review identified 25 studies examining decision-making among cosmetic and bariatric medical tourists from countries with universal healthcare. Key drivers for MT included ineligibility for domestic procedures and, for bariatric patients, long wait times. While cost was the most cited motivator, perceived expertise and availability of procedures sometimes matched or outweighed financial considerations. Once committed to pursuing MT, individuals frequently encountered ambiguous online information and a lack of support from friends, family, and local HCPs. In response, medical tourists placed high value on customer service and turned to social media and forums, where personal stories provided reassurance and confidence in their decision. Although perceived risk negatively influenced MT intentions, qualitative evidence suggests that emotional investment in the surgery often leads individuals to reframe or downplay perceived risks. The study identified numerous research gaps, including prospective studies, and quantitative research on the information seeking behaviour of prospective medical tourists (particularly for bariatric tourists), comparisons between decision-making by types of MT, and the role of emotional investment in shaping risk perception.

These findings should be interpreted with caution due to methodological limitations of the included studies. Qualitative studies often lacked depth in analysis and interpretation. Quantitative studies frequently relied on non-representative samples with potential response bias and reported descriptive data listing multiple-choice motivations, which did little to inform on the relative importance and prioritisation of each motivator for medical tourists’ decision-making. Finally, both included mixed methods studies lacked data triangulation. Importantly, despite the rich range of decision-making and health behaviour theories of potential relevance to MT, applied lenses were extremely narrow, both in terms of their overall range and the way in which they were applied. For example, work grounded in the TPB typically assessed subjective norms through the opinions of friends and family, overlooking the significant influence of online communities identified in qualitative findings; a gap that remains unaddressed in the existing quantitative research. Additionally, while medical tourists appear to conduct extensive research to make informed decisions, those who do so may also be more likely to participate in research or engage in online forums, introducing potential sampling bias and limiting the generalisability of current findings.

Motivations and decision-making for fertility tourism and less invasive dental procedure appear to be mostly comparable to the findings of this review [49]. Within a comparative qualitative study of medical tourists from the UK, decision-making around fertility tourism appeared to be similar to for bariatric tourism, in that it was often driven by availability, eligibility, and long waiting times within MT’s home countries, and medical tourists often relied on online and offline social networks for information [49]. However, motivations for MT for dental procedures appear to more greatly emphasise opportunity for vacation compared to bariatric and cosmetic tourism, and dissatisfaction and mistrust with UK private healthcare as opposed to emphasis on cost [49]. Additional motivators identified in the wider international MT literature that were absent in this review were ‘*push*’ motivators of desire for privacy and confidentiality of treatments [28], cultural similarities [28], and illegality of the procedure in the medical tourist’s home country [29], and ‘*pull*’ motivators of language proficiency of medical staff and accreditation [28]. Additionally, American medical tourists across types of MT appear to be influenced by the advice of physicians [29], which was not identified as an important factor in the current review.

### Recommendations and considerations for policy

Recommendations for home countries made within existing literature aim to deter cosmetic and bariatric tourism through prohibitive policies to reduce its appeal, such as limiting access to aftercare [11] or increasing legal liability for domestic providers [18], due to the risk of complications. However, the current review highlights that such approaches may be limited in effectiveness, as they overlook the strong emotional investment many medical tourists have in accessing potentially life-changing surgery at an affordable cost. This emotional commitment often leads medical tourists to downplay or dismiss risks, a behaviour consistent with cognitive dissonance theory, which suggests that individuals seek decision-consistent information to reduce psychological discomfort [71]. Commercial MT websites and facilitators exploit this tendency by presenting polished, emotionally appealing narratives that highlight positive outcomes and minimise risks, thereby reinforcing low-risk perceptions and strengthening intentions to travel [69, 72–74]. The unregulated, mainly provider-driven available information online makes it difficult for medical tourists to make informed decisions. In this context, harm reduction strategies that acknowledge medical tourists’ strong emotional drivers for surgery may prove more effective. Guidance from institutions such as the Royal College of Surgeons of England provides a useful starting point [75], but further efforts should address the difficulty medical tourists have in appraising unregulated and misleading information online.

In addition, harm reduction strategies could leverage the power of anecdotal evidence identified in the current review and wider literature [76], particularly through sharing peer narratives and patient experiences, whilst framing them with accurate, balanced information. Also, the current study found a lack of support from domestic HCPs for medical tourists, resulting in limited engagement and discussion during the decision-making process, which represents a missed opportunity to improve safe MT. Indeed, HCPs report challenges navigating discussions around MT with their patients [77]. HCPs discussed a reduced involvement in the decision making which led them to shift responsibility onto the patient, which, combined with HCP’s concerns about the quality of care abroad, could strain relationships [77]. Thus, guidance for HCPs in navigating these situations to foster a communication pathway would also be beneficial to reducing the harms of MT.

### Strengths and limitations

The main strength of the current review was the focus on medical tourists from countries with universal healthcare, which allowed for a meaningful synthesis and findings that were comparable and generalisable across such countries. Additionally, the inclusion of grey literature allowed for a comprehensive synthesis which included large and informative qualitative studies which would otherwise have been excluded. Another key strength was the incorporation of both qualitative and quantitative data in the review. Triangulation allowed for assessment of the extent to which the quantitative data validated the qualitative findings and allowed for identification of quantitative research gaps. However, the available quantitative data was limited, and three of the six included studies that applied inferential data analysis aimed to promote MT in the destination country rather than reduce its harms, which meant that the perspective was limited. Another limitation was the limited timeline of the review which meant that a rapid approach was taken, and independent data extraction was not conducted, with independent screening limited to a percentage of articles. However, screening reached good inter-rater reliability and extraction of study findings were checked by a second reviewer, in accordance with Cochrane guidance for rapid reviews [34]. Also, the current review focused on common high-risk forms of MT with limited or no universal healthcare coverage (bariatric and cosmetic) within the MT’s home country. Thus, the motivations and decision-making may differ for other types of MT, as discussed above. Lastly, only studies published in English language were included due to a lack of availability of translation services, which may have limited the comprehensiveness of the review. This limitation is particularly relevant given the international relevance of MT. A future, more expansive review that also included common destination countries for bariatric and cosmetic MT should aim to limit language restrictions to ensure the inclusion of literature from frequent destination countries such as Poland, Belgium, and Turkey [78].

## Conclusions

Individuals seeking cosmetic or bariatric surgery are inevitably drawn to the often-fractional price of surgery abroad, facilitated by increased international interconnectivity and reinforced by perceptions of higher perceived quality and ‘customer’ service. At this point, one intervention approach could be to highlight the total accumulated costs which can be higher than anticipated. Faced with insufficient reliable information online, prospective medical tourists seek advice from support networks, who are often found online due to judgement from friends and family. Personal stories are compelling and foster a dream of surgery abroad for the MT, leading them to downplay the risks. Clearly, international accreditation and regulation of surgeons is needed. Until then, interventions to reduce the harms of MT should utilise the power of anecdotal evidence to deliver accessible information that warns medical tourists of risk-masking tactics by providers and helps them to appraise information about risk. It is also crucial for domestic HCPs to receive support in navigating conversations with their patients around MT, to maintain an open and informed channel of communication so that medical tourists feel able to discuss their intentions and return for aftercare if needed. Prospective quantitative research is needed to explore medical tourists’ use of different information sources throughout their MT journey, and to assess the impact of emotional investment on risk perception and management in medical tourists, to validate the qualitative findings from the current review. Whilst in some cases deterring medical tourists may be possible, the appeal of lower cost surgery combined with the emotional investment in receiving the surgery means that the most impactful efforts to address the harms associated with MT may be to encourage safe decision-making and adherence to aftercare post-surgery.

## Supplementary Information

Below is the link to the electronic supplementary material.

Supplementary Material 1

## Data Availability

The full strategy applied to each database is available in Supplementary Material 3. The data used for this study is available in the full texts of included studies.

## References

[1] Hanefeld J, Horsfall D, Lunt N, Smith R. Medical tourism: a cost or benefit to the NHS? PLoS ONE. 2013;8(10):e70406. 24204556 10.1371/journal.pone.0070406 PMC3812100

[2] Lunt N. Medical tourism: treatments, markets and health system implications: a scoping review. Organisation for Economic Co-operation and Development. 2011.

[3] Pocock NS, Phua KH. Medical tourism and policy implications for health systems: a conceptual framework from a comparative study of Thailand, Singapore and Malaysia. Global Health. 2011;7:1–12. 21539751 10.1186/1744-8603-7-12 PMC3114730

[4] Whittaker A, Manderson L, Cartwright E. Patients without borders: understanding medical travel. Med Anthropol. 2010;29(4):336–43. 21082481 10.1080/01459740.2010.501318

[5] Lunt N, Green ST, Mannion R, Horsfall D. Quality, safety and risk in medical tourism. Medical Tourism: Routledge; 2012. pp. 31–46.

[6] Henry N, Abed H, Warner R. The ever-present costs of cosmetic surgery tourism: a 5-year observational study. Aesthetic Plast Surg. 2021;45:1912–9. 33625528 10.1007/s00266-021-02183-w

[7] Zuberi S, Egiz A, Iqbal H, Jambulingam P, Whitelaw D, Adil T, et al. Characterizing barriers and facilitators of metabolic bariatric surgery tourism: a systematic review. Br J Surg. 2024;111(3):znae060. 38547416 10.1093/bjs/znae060

[8] Lunt NT, Mannion R, Exworthy M. A framework for exploring the policy implications of UK medical tourism and international patient flows. Social Policy Adm. 2013;47(1):1–25.

[9] Gildea A, Shukla S, Parretti H, Khan O. Referral criteria and assessment for bariatric surgery: summary of updated NICE guidance. BMJ. 2023;382. 10.1136/bmj.p1880 37643791

[10] Bolckmans R, Askari A, Currie A, Ahmed AR, Batterham RL, Byrne J, et al. Clinical characteristics of patients undergoing primary bariatric surgery in the United Kingdom based on the National Bariatric Surgery Registry. Clin Obes. 2023;13(3):e12585. 36807508 10.1111/cob.12585

[11] Mahase E. Weight loss surgery: Patients need clearer warnings of risks from overseas care, say doctors. BMJ: Br Med J (Online). 2024;384:q583. 10.1136/bmj.q583 38453209

[12] England C, Bromham N, Needham-Taylor A, Hounsome J, Gillen E, Ingram B-J, et al. Complications and costs to the UK National Health Service due to outward medical tourism for elective surgery: a rapid review. BMJ open. 2026;16(1):e109050. 41529913 10.1136/bmjopen-2025-109050 PMC12815047

[13] Carey C, James S, Jaunoo S. A systematic review of patient and clinician experiences of bariatric tourism. The Annals of The Royal College of Surgeons of England. 2025. 10.1308/rcsann.2025.0020 PMC12949703 40197061

[14] Spurzem GJ, Ruiz-Cota P, Rocha A, Fontaine-Nicola A, Reyes E, Gabaldon K et al. Getting more than what you pay for? Managing complications of bariatric tourism at an academic center near the US-Mexico border. Surg Endosc. 2025:1–8. 10.1007/s00464-025-11850-x PMC12222374 40473949

[15] McCrossan S, Martin S, Hill C. Medical tourism in aesthetic breast surgery: a systematic review. Aesthetic Plast Surg. 2021;45:1895–909. 33876284 10.1007/s00266-021-02251-1 PMC8054849

[16] Brightman L, Ng S, Ahern S, Cooter R, Hopper I. Cosmetic tourism for breast augmentation: a systematic review. ANZ J Surg. 2018;88(9):842–7. 29205748 10.1111/ans.14326

[17] Foley BM, Haglin JM, Tanzer JR, Eltorai AE. Patient care without borders: a systematic review of medical and surgical tourism. J Travel Med. 2019;26(6):taz049. 31281926 10.1093/jtm/taz049

[18] Griffiths D, Mullock A. Cosmetic surgery: regulatory challenges in a global beauty market. Health Care Anal. 2018;26:220–34. 28247102 10.1007/s10728-017-0339-5 PMC6061011

[19] Kim DH, Sheppard CE, de Gara CJ, Karmali S, Birch DW. Financial costs and patients’ perceptions of medical tourism in bariatric surgery. Can J Surg. 2016;59(1):59–61. 26574702 10.1503/cjs.004215 PMC4734921

[20] Farid M, Nikkhah D, Little M, Edwards D, Needham W, Shibu M. Complications of cosmetic surgery abroad–cost analysis and patient perception. Plast Reconstr Surgery–Global Open. 2019;7(6):e2281. 10.1097/GOX.0000000000002281 PMC6635218 31624684

[21] Rafeh S, Michael F, Amy G, Elaf O, Paul R, James OR, et al. An analysis of the cost and impact of cosmetic tourism and its associated complications: a multi institutional study. Surgeon. 2022;20(6):339–44. 35012867 10.1016/j.surge.2021.12.007

[22] Livingston R, Berlund P, Eccles-Smith J, Sawhney R. The real cost of cosmetic tourism cost analysis study of cosmetic tourism complications presenting to a public hospital. Eplasty. 2015;15:e34. 26240672 PMC4522144

[23] Sheppard CE, Lester EL, Karmali S, de Gara CJ, Birch DW. The cost of bariatric medical tourism on the Canadian healthcare system. Am J Surg. 2014;207(5):743–7. 24791638 10.1016/j.amjsurg.2014.01.004

[24] Sheppard CE, Lester EL, Chuck AW, Kim DH, Karmali S, de Gara CJ, Birch DW. Medical tourism and bariatric surgery: who pays? Surg Endosc. 2014;28(12):3329–36. 24969849 10.1007/s00464-014-3613-8

[25] McGirr J, Gregg EW, McNamara DA, O’Malley G. Bariatric and metabolic surgery medical tourism: the compelling need for regulation through transnational collaboration. BMJ Global Health. 2025;10(7). 10.1136/bmjgh-2025-019546 PMC12273068 40664433

[26] Dobbie LJ, Birney S, Breen C, Bryant S, Clare K, Ciudin A, et al. European recommendations from healthcare professionals and people living with obesity on safe practice for bariatric and metabolic surgery medical tourism: a modified Delphi consensus statement from EASO, IFSO-EC, and ECPO. Int J Surg. 2025;111(2):1711–23. 39705126 10.1097/JS9.0000000000002171

[27] England C, Needham-Taylor A, Bromham N, Hounsome J, Gillen E, Davies JR et al. Complications and costs to the NHS due to outward medical tourism for elective surgery: a rapid review. medRxiv. 2025:2025.04. 02.25325086. 10.1136/bmjopen-2025-109050 PMC12815047 41529913

[28] John SP, Larke R. An analysis of push and pull motivators investigated in medical tourism research published from 2000 to 2016. Tourism Rev Int. 2016;20(2–3):73–90.

[29] Xu T, Wang W, Du J. An integrative review of patients’ experience in the medical tourism. INQUIRY: J Health Care Organ Provis Financing. 2020;57:0046958020926762. 10.1177/0046958020926762 PMC7285947 32513038

[30] Majeed S, Lu C. Changing preferences, moving places and third party administrators: a scoping review of medical tourism trends (1990–2016). Almatourism-Journal Tourism Cult Territorial Dev. 2017;8(15):56–83.

[31] Najafi B, Raeissi P, Gorji H, Ahmadi A, Haghighi M. Identifying factors affecting destination choice of medical tourists: a systematic review of literature. J Fundamental Appl Sci. 2017;9(2S):1309–28.

[32] Crooks VA, Kingsbury P, Snyder J, Johnston R. What is known about the patient’s experience of medical tourism? A scoping review. BMC Health Serv Res. 2010;10:1–12. 20825667 10.1186/1472-6963-10-266 PMC2944273

[33] Page MJ, McKenzie JE, Bossuyt PM, Boutron I, Hoffmann TC, Mulrow CD et al. The PRISMA 2020 statement: an updated guideline for reporting systematic reviews. BMJ. 2021;372. 10.1136/bmj.n71 PMC8005924 33782057

[34] Garritty C, Hamel C, Trivella M, Gartlehner G, Nussbaumer-Streit B, Devane D et al. Updated recommendations for the Cochrane rapid review methods guidance for rapid reviews of effectiveness. BMJ. 2024;384. 10.1136/bmj-2023-076335 38320771

[35] Stevens A, Hersi M, Garritty C, Hartling L, Shea BJ, Stewart LA, et al. Rapid review method series: interim guidance for the reporting of rapid reviews. BMJ evidence-based Med. 2025;30(2):118–23. 10.1136/bmjebm-2024-112899 PMC12013547 39038926

[36] Cooke A, Smith D, Booth A. Beyond PICO: the SPIDER tool for qualitative evidence synthesis. Qual Health Res. 2012;22(10):1435–43. 22829486 10.1177/1049732312452938

[37] World Health Organization. Universal health coverage (UHC) 2025 [Available from: https://www.who.int/news-room/fact-sheets/detail/universal-health-coverage-(uhc)

[38] McHugh ML. Interrater reliability: the kappa statistic. Biochemia Med. 2012;22(3):276–82. PMC3900052 23092060

[39] Altman DG. Practical statistics for medical research. Chapman and Hall/CRC; 1990.

[40] Noyes J, Booth A, Flemming K, Garside R, Harden A, Lewin S, et al. Cochrane Qualitative and Implementation Methods Group guidance series—paper 3: methods for assessing methodological limitations, data extraction and synthesis, and confidence in synthesized qualitative findings. J Clin Epidemiol. 2018;97:49–58. 29247700 10.1016/j.jclinepi.2017.06.020

[41] Cochrane Effective Practice and Organisation of Care (EPOC). Good practice data extraction form 2017 [Available from: https://epoc.cochrane.org/resources/epoc-resources-review-authors

[42] Hong QN, Fàbregues S, Bartlett G, Boardman F, Cargo M, Dagenais P, et al. The Mixed Methods Appraisal Tool (MMAT) version 2018 for information professionals and researchers. Educ Inform. 2018;34(4):285–91.

[43] Thomas J, Harden A. Methods for the thematic synthesis of qualitative research in systematic reviews. BMC Med Res Methodol. 2008;8:1–10. 18616818 10.1186/1471-2288-8-45 PMC2478656

[44] Campbell M, McKenzie JE, Sowden A, Katikireddi SV, Brennan SE, Ellis S et al. Synthesis without meta-analysis (SWiM) in systematic reviews: reporting guideline. BMJ. 2020;368. 10.1136/bmj.l6890 PMC7190266 31948937

[45] Tashakkori A, Teddlie C. Mixed methodology: Combining qualitative and quantitative approaches: sage; 1998.

[46] Thomas J, Harden A, Oakley A, Oliver S, Sutcliffe K, Rees R, et al. Integrating qualitative research with trials in systematic reviews. BMJ. 2004;328(7446):1010–2. 15105329 10.1136/bmj.328.7446.1010 PMC404509

[47] Baán R. Look what you made me do: Social media influencers’ impact on medical travel decisions. ISCTE-Instituto Universitario de Lisboa (Portugal); 2023.

[48] Chia KW, Liao YM. An Exploratory Study of Factors Influencing Chinese Outbound Medical Tourism. J China Tourism Res. 2021;17(3):376–94.

[49] Hanefeld J, Lunt N, Smith R, Horsfall D. Why do medical tourists travel to where they do? The role of networks in determining medical travel. Soc Sci Med. 2015;124:356–63. 24976006 10.1016/j.socscimed.2014.05.016

[50] Holliday R, Bell D, Jones M, Probyn E, Sanchez Taylor J. Sun, sea, sand and silicone: mapping cosmetic surgery tourism. 2014.

[51] Jackson C, Snyder J, Crooks VA, Lavergne MR. I didn’t have to prove to anybody that I was a good candidate: a case study framing international bariatric tourism by Canadians as circumvention tourism. BMC Health Serv Res. 2018;18. 10.1186/s12913-018-3385-2 PMC6053718 30029651

[52] Jackson C, Snyder J, Crooks VA, Lavergne M. Exploring isolation, self-directed care and extensive follow-up: Factors heightening the health and safety risks of bariatric surgery abroad among Canadian medical tourists. Int J Qual Stud Health Well-being. 2019;14(1). ArtID 1613874. 10.1080/17482631.2019.1613874 PMC6522967 31084487

[53] Abd Mutalib NS, Soh YC, Wong TW, Yee SM, Yang Q, Murugiah MK, Ming LC. Online narratives about medical tourism in Malaysia and Thailand: a qualitative content analysis. J Travel Tourism Mark. 2017;34(6):821–32.

[54] Noaman S, Montargot N, Understanding interactions between providers of cosmetic tourism, and tourists: the lebanon experience. Tourism Cult Communication. 2023;23(1):45–60.

[55] Ozan-Rafferty ME, Johnson JA, Shah GH, Kursun A. In the words of the medical tourist: an analysis of Internet narratives by health travelers to Turkey. J Med Internet Res. 2014;16(2):e2694. 10.2196/jmir.2694 PMC3936263 24513565

[56] Prasad T, Forsyth R. Cosmetic medical tourists’ use of online and offline experiential and procedural information resources in decision-making: Implications for digital health literacy and neoliberalism. Qualitative Health Communication.3(2):72–90.

[57] Robertson EM, Moorman SWJ, Korus LJ. Why Do Canadians Travel Abroad for Cosmetic Surgery? A Qualitative Analysis on Motivations for Cosmetic Surgery Tourism. Plast Surg. 2022;30(4):353–9. 10.1177/22925503211019607 PMC9537712 36212104

[58] Jones M, Bell D, Holliday R, Probyn E, Taylor JS. Facebook and facelifts: Communities of cosmetic surgery tourists. Travel and transformation: Routledge; 2016. pp. 189–204.

[59] Anar EN, Kırtıl İ. Relationship between preoperative surgical fear, anxiety, and satisfaction levels in individuals choosing bariatric surgery tourism: A descriptive, cross-sectional study. Obes Surg. 2025:1–11. 10.1007/s11695-025-07749-0 PMC11976799 40053303

[60] Arrobas F. Dental tourism: How to promote Lisbon as a destination for the English population? 2021.

[61] Carmagnola D, Filippucci L, Celestino S, Carrassi A, Delia S, Lodi G. A survey on the experience with dental tourism in a sample of Italian patients. Minerva Stomatol. 2012;61(1–2):11–20. 22274306

[62] Lee M, Han H, Lockyer T, Medical tourism-attracting Japanese, tourists for medical tourism experience. J Travel Tourism Mark. 2012;29(1):69–86.

[63] Liang LJ, Choi HC, Joppe M, Lee W. Examining medical tourists’ intention to visit a tourist destination: Application of an extended MEDTOUR scale in a cosmetic tourism context. Int J Tourism Res. 2019;21(6):772–84.

[64] Majeed S, Zhou ZM, Ramkissoon H. Beauty and elegance: value co-creation in cosmetic surgery tourism. Sage Open. 2020;10(2).

[65] Martin S, Long R, Hill C, Sinclair S. Cosmetic Tourism in Northern Ireland. Ann Plast Surg. 2019;83(6):618–21. 31688106 10.1097/SAP.0000000000002081

[66] Nam H-m. Perceived risk of cosmetic surgery tourism: scale development and its application in segmenting Chinese cosmetic surgery tourists. 2020.

[67] Nassab R, Hamnett N, Nelson K, Kaur S, Greensill B, Dhital S, Juma A. Cosmetic tourism: public opinion and analysis of information and content available on the Internet. Aesthetic Surg J. 2010;30(3):465–9. 10.1177/1090820X10374104 20601579

[68] Whiteman E, Romain K, Welman T, Mitchell C, Gabuniya N, Collins D, Markeson D. The rising NHS burden from cosmetic surgery procedures performed abroad and non-surgical procedures performed in the United Kingdom. J Plast Reconstr Aesthetic Surg. 2025;102:39–41. 10.1016/j.bjps.2025.01.017 39892185

[69] Majeed S, Kim WG, Ryu K. Medical Tourism and Cognitive Dissonance: Exploring Tourist Choice Behavior, Post-Choice Pre-Outcome Regret, and Visit intention. J Qual Assur Hospitality Tourism. 2024;25(3):514–44.

[70] Thayarnsin SL-o-i. The role of risk, culture, image and quality on destination loyalty: Perspectives from international medical tourists toward Thailand as a medical tourism destinationole of risk, culture, image and quality on destination loyalty: Perspectives from international medical tourists toward Thailand as a medical tourism destination. Dissertation Abstracts International: Sect B: Sci Eng. 2023;84(8–B):NoPaginationSpecified.

[71] Harmon-Jones E, Mills J. An introduction to cognitive dissonance theory and an overview of current perspectives on the theory. 2019.

[72] McMahon M, Gressmann K, Martin-Smith J. An objective analysis of quality and readability of online information for patients seeking cosmetic surgery abroad. J Plast Reconstr Aesthetic Surg. 2023;81:88–90. 10.1016/j.bjps.2023.04.051 37121048

[73] Penney K, Snyder J, Crooks VA, Johnston R. Risk communication and informed consent in the medical tourism industry: a thematic content analysis of Canadian broker websites. BMC Med Ethics. 2011;12:1–9. 21943392 10.1186/1472-6939-12-17 PMC3189886

[74] Mason A, Wright KB. Framing medical tourism: an examination of appeal, risk, convalescence, accreditation, and interactivity in medical tourism web sites. J health communication. 2011;16(2):163–77. 21161812 10.1080/10810730.2010.535105

[75] Royal College of Surgeons of England. Thinking of having cosmetic surgery abroad? n.d [Available from: https://www.rcseng.ac.uk/patient-care/cosmetic-surgery/having-surgery-abroad/

[76] Rothchild E, Chernovolenko D, Wang F, Ricci JA. An analysis of male plastic surgery content on TikTok. Aesthetic Surg J. 2024;44(5):556–64. 10.1093/asj/sjad350 37972242

[77] Snyder J, Crooks VA, Johnston R. Perceptions of the Ethics of Medical Tourism: Comparing Patient and Academic Perspectives. Public Health Ethics. 2012;5(1):38–46.

[78] Border P. Ouward medical tourism. 2020.

