# Supplementary material for: Decision-making about bariatric and cosmetic medical tourism from countries with universal healthcare: a rapid systematic review
Source: Global Health. 2026 Mar 28;22:45. doi: 10.1186/s12992-026-01207-x (PMC13151202; doi:10.1186/s12992-026-01207-x)
Supplement: Supplementary file 1 — Supplementary Material 1 [file 12992_2026_1207_MOESM1_ESM.docx]

Supplementary Material 2: Inclusion and exclusion criteria according to the SPIDER framework.

| **SPIDER framework** | **Included** | **Excluded** |
| --- | --- | --- |
| Sample | - Medical tourists of all age ranges, genders, and ethnicities - Previous, prospective, or potential medical tourists: those who have engaged in medical tourism, have planned to, or considered it - Studies that compare the decision making of previous, prospective, or potential medical tourists with participants that have not considered medical tourism and choose the same medical treatment within the UK - Outbound from countries with universal healthcare that is either free (e.g. UK, Norway, Sweden, Denmark, Spain, Italy, Finland, Canada, Australia, New Zealand, South Korea, Brazil) or affordable with mandatory health insurance (e.g. Germany, France, Japan) | - Countries with private healthcare systems (with no free universal healthcare available) e.g. USA - Inbound tourists to the UK - Participants involved in or affected by medical tourism but not decision-making for themselves (e.g. surgeons, nurses, or family and friends of previous, prospective, or potential medical tourists) |
| Phenomenon of interest | - Medical tourism: patients choosing to travel abroad for invasive medical procedures with limited or no NHS eligibility: dentistry (e.g. dental implants, veneers), weight loss/bariatric surgery (e.g. gastric bypass, gastric banding), or cosmetic (e.g. liposuction, BBL) - Knowledge, beliefs, motivations, factors predicting decision/intention - Revisiting/loyalty | - Patients who have been refereed abroad through cross-border care arrangements - Complementary and alternative care - Other types of invasive medical procedure e.g. fertility treatment, gender reassignment, organ transplants, cancer treatment, orthopedic surgery, cardiology/cardiac surgery, and stem cell therapy - Demographic predictors |
| Design | Primary research (qualitative or quantitative) | - Reviews (e.g. systematic, scoping) - Prevalence studies |
| Evaluation | Decision making process: why people chose to (or not) engage in medical tourism and what factors influence their decision-making | Studies merely investigating the prevalence of medical tourism and its complications |
| Research type | - Peer reviewed studies and grey literature (e.g. preprints, reports) - Any year of publication | Opinion pieces, commentaries, media coverage, book reviews, conference abstracts, literature reviews, and editorials |
| Language | English language | Non-English language |
| Databases | MEDLINE, PsycINFO, and Web of Science, Overton (for grey literature) | |

Supplementary Material 3: Full search strategy for each database searched

Supplementary Material 3a: Web of science search

4 searches: title (first search- medical tourism phrases), abstract (first search- medical tourism phrases), title (second search- pairing terms for MT), abstract (second search- pairing terms for MT):

First search- medical tourism phrases (title) (277 results)

(TI=(“Medical tourism” OR “Hospital outshopping” OR “Health tourism” OR “Cosmetic tourism” OR “Aesthetic tourism” OR “Overseas treatment” OR “Treatment overseas”)) AND TI=(Decision* OR Factors OR Attitude* OR Perspective* OR Motivat* OR Choice OR Ch$ose OR Concept* OR experience* OR attitude* OR perception* OR belief* OR opinion* OR view* OR perspective* OR voice* OR value* OR Story OR Stories OR Storytelling OR choice* OR account* OR reason* OR themes OR thematic OR narration OR "exploratory research" OR Theor* OR Intention* OR “accounts” OR “an account” OR Ethnograph* OR Autoethnography OR Benefit* )

First search- medical tourism phrases (abstract) (1192 results)

(AB=(“Medical tourism” OR “Hospital outshopping” OR “Health tourism” OR “Cosmetic tourism” OR “Aesthetic tourism” OR “Overseas treatment” OR “Treatment overseas”)) AND AB=(Decision* OR Factors OR Attitude* OR Perspective* OR Motivat* OR Choice OR Ch$ose OR Concept* OR experience* OR attitude* OR perception* OR belief* OR opinion* OR view* OR perspective* OR voice* OR value* OR Story OR Stories OR Storytelling OR choice* OR account* OR reason* OR themes OR thematic OR narration OR "exploratory research" OR Theor* OR Intention* OR “accounts” OR “an account” OR Ethnograph* OR Autoethnography OR Benefit* )

Second search- pairing terms for MT (Title) (30 results)

((TI=(Abroad OR Outsourcing OR Touris* OR Transnational OR Overseas OR “Cross$border”)) AND TI=(Dental OR dentist* OR bariatric OR “gastric sleeve” OR “vertical sleeve gastrectom*” OR “gastrectom* sleeve*” OR “sleeve gastrectom*” OR “gastric bypass” OR “stomach bypass” or "roux-en-y" OR “RYGB” OR “biliopancreatic bypass” OR “duodenal switch*” OR “duodenum switch*” OR “biliopancreatic diver*” OR “gastric restrict*” OR “gastric band*” OR lap$band* OR “laparoscop* band*” OR “gastric balloon” OR “brow lift” OR Face$lift OR “Neck lift” OR “breast augmentation” OR “Breast implants” OR “Breast lift” OR “Brazilian Butt Lift” OR liposuction OR “patient mobility” OR “Rhinoplasty” OR “Rhytidoplasty” OR “Genioplasty” OR “otoplasty” OR “Blepharoplasty” OR “abdominoplasty” OR “tummy tuck” OR “Plastic surgery” OR lipoinjection OR “Buttock* augmentation” OR “Buttock* enhancement” OR “Gluteal fat grafting” OR “Buttock* implants” OR “Esthetic surgery” OR “Aesthetic surgery” OR “Body contouring surgery”)) AND TI=(Decision* OR Factors OR Attitude* OR Perspective* OR Motivat* OR Choice OR Ch$ose OR Concept* OR experience* OR attitude* OR perception* OR belief* OR opinion* OR view* OR perspective* OR voice* OR value* OR Story OR Stories OR Storytelling OR choice* OR account* OR reason* OR themes OR thematic OR narration OR "exploratory research" OR Theor* OR Intention* OR “accounts” OR “an account” OR Ethnograph* OR Autoethnography OR Benefit* )

Second search- pairing terms for MT (abstract) (345 results)

((AB=(Abroad OR Outsourcing OR Touris* OR Transnational OR Overseas OR “Cross$border”)) AND AB=(Dental OR dentist* OR bariatric OR “gastric sleeve” OR “vertical sleeve gastrectom*” OR “gastrectom* sleeve*” OR “sleeve gastrectom*” OR “gastric bypass” OR “stomach bypass” or "roux-en-y" OR “RYGB” OR “biliopancreatic bypass” OR “duodenal switch*” OR “duodenum switch*” OR “biliopancreatic diver*” OR “gastric restrict*” OR “gastric band*” OR lap$band* OR “laparoscop* band*” OR “gastric balloon” OR “brow lift” OR Face$lift OR “Neck lift” OR “breast augmentation” OR “Breast implants” OR “Breast lift” OR “Brazilian Butt Lift” OR liposuction OR “patient mobility” OR “Rhinoplasty” OR “Rhytidoplasty” OR “Genioplasty” OR “otoplasty” OR “Blepharoplasty” OR “abdominoplasty” OR “tummy tuck” OR “Plastic surgery” OR lipoinjection OR “Buttock* augmentation” OR “Buttock* enhancement” OR “Gluteal fat grafting” OR “Buttock* implants” OR “Esthetic surgery” OR “Aesthetic surgery” OR “Body contouring surgery”)) AND AB=(Decision* OR Factors OR Attitude* OR Perspective* OR Motivat* OR Choice OR Ch$ose OR Concept* OR experience* OR attitude* OR perception* OR belief* OR opinion* OR view* OR perspective* OR voice* OR value* OR Story OR Stories OR Storytelling OR choice* OR account* OR reason* OR themes OR thematic OR narration OR "exploratory research" OR Theor* OR Intention* OR “accounts” OR “an account” OR Ethnograph* OR Autoethnography OR Benefit* )

Supplementary Material 3b: Medline: (Ovid MEDLINE® ALL) search

First search (medical tourism phrases): (341 results)

1. (("Medical tourism" or "Hospital outshopping" or "Health tourism" or "Cosmetic tourism" or "Aesthetic tourism" or "Overseas treatment" or "Treatment overseas") and (Decision* or Factors or Attitude* or Perspective* or Motivat* or Choice or Ch?ose or Concept* or experience* or attitude* or perception* or belief* or opinion* or view* or perspective* or voice* or value* or Story or Stories or Storytelling or choice* or account* or reason* or themes or thematic or narration or "exploratory research" or Theor* or Intention* or "accounts" or "an account" or Ethnograph* or Autoethnography or Benefit*)).ti,ab.
2. limit 1 to (human and english language)
3. 1 and 2

Second search (pairing terms for MT): (272 results)

1. ((Abroad or Outsourcing or Touris* or Transnational or Overseas or "Cross?border") and (Dental or dentist* or bariatric or "gastric sleeve" or "vertical sleeve gastrectom*" or "gastrectom* sleeve*" or "sleeve gastrectom*" or "gastric bypass" or "stomach bypass" or "roux-en-y" or "RYGB" or "biliopancreatic bypass" or "duodenal switch*" or "duodenum switch*" or "biliopancreatic diver*" or "gastric restrict*" or "gastric band*" or lap?band* or "laparoscop* band*" or "gastric balloon" or "brow lift" or Face?lift or "Neck lift" or "breast augmentation" or "Breast implants" or "Breast lift" or "Brazilian Butt Lift" or liposuction or "patient mobility" or "Rhinoplasty" or "Rhytidoplasty" or "Genioplasty" or "otoplasty" or "Blepharoplasty" or "abdominoplasty" or "tummy tuck" or "Plastic surgery" or lipoinjection or "Buttock* augmentation" or "Buttock* enhancement" or "Gluteal fat grafting" or "Buttock* implants" or "Esthetic surgery" or "Aesthetic surgery" or "Body contouring surgery") and (Decision* or Factors or Attitude* or Perspective* or Motivat* or Choice or Ch?ose or Concept* or experience* or attitude* or perception* or belief* or opinion* or view* or perspective* or voice* or value* or Story or Stories or Storytelling or choice* or account* or reason* or themes or thematic or narration or "exploratory research" or Theor* or Intention* or "accounts" or "an account" or Ethnograph* or Autoethnography or Benefit*)).ti,ab.
2. limit 1 to (human and english language)
3. 1 and 2

Supplementary Material 3c: Psychinfo: (from 1967) search

First search (medical tourism phrases): (172 results)

1. (("Medical tourism" or "Hospital outshopping" or "Health tourism" or "Cosmetic tourism" or "Aesthetic tourism" or "Overseas treatment" or "Treatment overseas") and (Decision* or Factors or Attitude* or Perspective* or Motivat* or Choice or Ch?ose or Concept* or experience* or attitude* or perception* or belief* or opinion* or view* or perspective* or voice* or value* or Story or Stories or Storytelling or choice* or account* or reason* or themes or thematic or narration or "exploratory research" or Theor* or Intention* or "accounts" or "an account" or Ethnograph* or Autoethnography or Benefit*)).ti,ab.
2. limit 1 to (human and english language)
3. 1 and 2

Second search (pairing terms for MT): (35 results)

1. ((Abroad or Outsourcing or Touris* or Transnational or Overseas or "Cross?border") and (Dental or dentist* or bariatric or "gastric sleeve" or "vertical sleeve gastrectom*" or "gastrectom* sleeve*" or "sleeve gastrectom*" or "gastric bypass" or "stomach bypass" or "roux-en-y" or "RYGB" or "biliopancreatic bypass" or "duodenal switch*" or "duodenum switch*" or "biliopancreatic diver*" or "gastric restrict*" or "gastric band*" or lap?band* or "laparoscop* band*" or "gastric balloon" or "brow lift" or Face?lift or "Neck lift" or "breast augmentation" or "Breast implants" or "Breast lift" or "Brazilian Butt Lift" or liposuction or "patient mobility" or "Rhinoplasty" or "Rhytidoplasty" or "Genioplasty" or "otoplasty" or "Blepharoplasty" or "abdominoplasty" or "tummy tuck" or "Plastic surgery" or lipoinjection or "Buttock* augmentation" or "Buttock* enhancement" or "Gluteal fat grafting" or "Buttock* implants" or "Esthetic surgery" or "Aesthetic surgery" or "Body contouring surgery") and (Decision* or Factors or Attitude* or Perspective* or Motivat* or Choice or Ch?ose or Concept* or experience* or attitude* or perception* or belief* or opinion* or view* or perspective* or voice* or value* or Story or Stories or Storytelling or choice* or account* or reason* or themes or thematic or narration or "exploratory research" or Theor* or Intention* or "accounts" or "an account" or Ethnograph* or Autoethnography or Benefit*)).ti,ab.
2. limit 1 to (human and english language)
3. 1 and 2

Supplementary Material 3d: Overton search

(17 results)

Abstract: (("Medical tourism" OR "Hospital outshopping" OR "Health tourism" OR "Cosmetic tourism" OR "Aesthetic tourism" OR "Overseas treatment" OR "Treatment overseas") AND (Decision* OR Factors OR Attitude* OR Perspective* OR Motivat* OR Choice OR Chose OR Choose OR Concept* OR experience* OR attitude* OR perception* OR belief* OR opinion* OR view* OR perspective* OR voice* OR value* OR Story OR Stories OR Storytelling OR choice* OR account* OR reason* OR themes OR thematic OR narration OR "exploratory research" OR Theor* OR Intention* OR "accounts" OR "an account" OR Ethnograph* OR Autoethnography OR Benefit*))

(24 results)

Abstract: ((Abroad OR Outsourcing OR Touris* OR Transnational OR Overseas OR "Cross-border" OR “Cross border”) AND (Dental OR dentist* OR bariatric OR "gastric sleeve" OR "vertical sleeve gastrectom*" OR "gastrectom* sleeve*" OR "sleeve gastrectom*" OR "gastric bypass" OR "stomach bypass" OR "roux-en-y" OR "RYGB" OR "biliopancreatic bypass" OR "duodenal switch*" OR "duodenum switch*" OR "biliopancreatic diver*" OR "gastric restrict*" OR "gastric band*" OR “lap band*” OR lapband* OR lap-band* OR "laparoscop* band*" OR "gastric balloon" OR "brow lift" OR Facelift OR “Face lift” OR Face-lift OR "Neck lift" OR "breast augmentation" OR "Breast implants" OR "Breast lift" OR "Brazilian Butt Lift" OR liposuction OR "patient mobility" OR "Rhinoplasty" OR "Rhytidoplasty" OR "Genioplasty" OR "otoplasty" OR "Blepharoplasty" OR "abdominoplasty" OR "tummy tuck" OR "Plastic surgery" OR lipoinjection OR "Buttock* augmentation" OR "Buttock* enhancement" OR "Gluteal fat grafting" OR "Buttock* implants" OR "Esthetic surgery" OR "Aesthetic surgery" OR "Body contouring surgery") AND (Decision* OR Factors OR Attitude* OR Perspective* OR Motivat* OR Choice OR Chose OR Choose OR Concept* OR experience* OR attitude* OR perception* OR belief* OR opinion* OR view* OR perspective* OR voice* OR value* OR Story OR Stories OR Storytelling OR choice* OR account* OR reason* OR themes OR thematic OR narration OR "exploratory research" OR Theor* OR Intention* OR "accounts" OR "an account" OR Ethnograph* OR Autoethnography OR Benefit*))

Supplementary Material 4: Bespoke data extraction form items. Each bullet point was an extracted column in an excel file.

- Study reference (First author, Date)
- Study title
- Initials of person extracting
- Initials of person who reviewed and verified the results
- Funding source
- Conflict of interest
- Study aim
- Design
- Sample size (of relevant sample)
- Participants (Sampling type and recruitment sources, data collection period, location and context, inclusion/exclusion criteria)
- Materials (brief summary of relevant materials)
- Procedure (brief summary if relevant)
- Data analysis type
- Relevant participant characteristics
- IF QUALITATIVE: number of quotes extracted
- IF QUALITATIVE: quotes
- IF QUALITATIVE: relevant themes and descriptions/explanations provided by study authors
- IF QUALITATIVE: author conclusions
- IF QUANTITATIVE: results of quantitative analyses E.g. any significant or non-significant findings, interactions reported)
- IF APPLIED THEORY: Application of theory (the theory used, how it was proposed to fit with the topic of medical tourism, and the utility of its application in this context)
- Relevant conclusions of study authors
- Relevant limitations
- Any correspondence with study authors
- Initials of first reviewer of quality appraisal
- Initials of independent review of quality appraisal

Supplementary Material 5: Quality appraisal of included quantitative (5a), qualitative (5b), and mixed methods (5c) studies.

Supplementary Material 5a: Quality of included qualitative studies

| Item | S1: Are there clear research questions? | S2: Do the collected data allow to address the research questions? | 1: Is the qualitative approach appropriate to answer the research question? | 2: Are the qualitative data collection methods adequate to address the research question? | 3: Are the findings adequately derived from the data? | 4: Is the interpretation of results sufficiently substantiated by data? | 5: Is there coherence between qualitative data sources, collection, analysis and interpretation? |
| --- | --- | --- | --- | --- | --- | --- | --- |
| Study | | | | | | | |
| Baan 2023 | Yes | Yes | Yes | Yes | No | No | No |
| Chia 2021 | Yes | Yes | Yes | Can’t tell | Can’t tell | Yes | Yes |
| Hanefeld 2015 | Yes | Yes | Yes | Yes | Yes | No | Yes |
| Holliday | Yes | Yes | Yes | Can’t tell | Can’t tell | Yes | Can’t tell |
| Jackson 2018 | Yes | Yes | Yes | Yes | No | Yes | Yes |
| Jackson 2019 | Yes | Yes | Yes | Yes | No | No | Yes |
| Mutalib 2017 | No | Yes | Yes | Yes | No | No | Yes |
| Noaman 2023 | Yes | Yes | Yes | Yes | No | Yes | No |
| Ozan-Rafferty 2014 | Yes | Yes | Yes | Yes | Yes | No | Yes |
| Prasad 2024 | Yes | Yes | Yes | Yes | Yes | Yes | Yes |
| Robertson 2022 | Yes | Yes | Yes | Can’t tell | Can’t tell | No | No |

Supplementary Material 5b: Quality of included descriptive quantitative studies. The items for ‘descriptive’ quantitative studies were employed for all included quantitative studies.

| Item | S1: Are there clear research questions? | S2: Do the collected data allow to address the research questions? | 1: Is the sampling strategy relevant to address the research question? | 2: Is the sample representative of the target population? | 3: Are the measurements appropriate? | 4: Is the risk of nonresponse bias low? | 5: Is the statistical analysis appropriate to answer the research question? |
| --- | --- | --- | --- | --- | --- | --- | --- |
| Study | | | | | | | |
| Anar 2025 | Yes | Yes | Yes | Can’t tell | Yes | Can’t tell | Yes |
| Arrobas 2021 | Yes | Yes | Yes | Yes | Yes | Can’t tell | Yes |
| Carmoagnola 2012 | Yes | Can’t tell | No | Can’t tell | No | Yes | Yes |
| Kim 2016 | Yes | Can’t tell | Can’t tell | No | Can’t tell | Can’t tell | Yes |
| Lee 2012 | Yes | Yes | Yes | Can’t tell | Yes | Can’t tell | Yes |
| Liang 2019 | Yes | Can’t tell | Yes | Yes | Yes | Yes | Yes |
| Majeed 2020 | Yes | Yes | Yes | Can’t tell | No | Can’t tell | Yes |
| Martin 2019 | Yes | Can’t tell | Yes | No | Can’t tell | Yes | No |
| Nam 2020 | Yes | Yes | Can’t tell | Can’t tell | Yes | Yes | Yes |
| Nassab 2010 | Yes | Can’t tell | Can’t tell | No | Can’t tell | Yes | Yes |
| Whiteman 2025 | Yes | Yes | Can’t tell | No | No | Can’t tell | Yes |

Supplementary Material 5c: Quality of included mixed methods studies. Explanation of each item is provided below.

| Item | S1 | S2 | 1 (qual) | 2  (qual) | 3  (qual) | 4  (qual) | 5  (qual) | 1  (quant) | 2  (quant) | 3  (quant) | 4  (quant) | 5  (quant) | 1  (mix) | 2  (mix) | 3  (mix) | 4  (mix) | 5  (mix) |
| --- | --- | --- | --- | --- | --- | --- | --- | --- | --- | --- | --- | --- | --- | --- | --- | --- | --- |
| Study | | | | | | | | | | | | | | | | | |
| Majeed 2024 | Yes | Yes | No | No | No | No | No | CT | CT | Yes | CT | Yes | Yes | No | No | No | No |
| Thayarnsin 2023 | Yes | Yes | CT | Yes | Yes | Yes | Yes | Yes | CT | No | No | Yes | CT | No | No | CT | No |

*Qual = items to assess the quality of the qualitative data collection. Items re the same as the quality appraisal for qualitative studies (see above). Quant = items to assess the quantitative data collection. Both studies collected descriptive quantitative data, so the items are the same as the quality appraisal for included quantitative studies (see above). Mix = items to assess the quality of the mixed methods design and analysis. CT = ‘Can’t tell’

- S1: Are there clear research questions?
- S2: Do the collected data allow to address the research questions?
- 1 (mix): Is there an adequate rationale for using a mixed methods design to address the research question?
- 2 (mix): Are the different components of the study effectively integrated to answer the research question?
- 3 (mix): Are the outputs of the integration of qualitative and quantitative components adequately interpreted?
- 4 (mix): Are divergences and inconsistencies between quantitative and qualitative results adequately addressed?
- 5 (mix): Do the different components of the study adhere to the quality criteria of each tradition of the methods involved?

Supplementary Material 6: Excluded articles based on full-text screening and reason for exclusion.

| **Reference** | **Reason for exclusion** |
| --- | --- |
| 1. Abbaspour, F., Soltani, S., & Tham, A. (2023). COVID-19 and medical tourism intentions for Iran–a test of the risk perception attitude framework (RPAF). Journal of Islamic Marketing, 14(6), 1506-1530. | Wrong patient population |
| 1. Abdul-Rahman, M. N., Hassan, T. H., Abdou, A. H., Abdelmoaty, M. A., Saleh, M. I., & Salem, A. E. (2023). Responding to tourists’ intentions to revisit medical destinations in the post-COVID-19 era through the promotion of their clinical trust and well-being. Sustainability, 15(3), 2399. | Wrong patient population |
| 1. Abubakar, A. M., & Ilkan, M. (2016). Impact of online WOM on destination trust and intention to travel: A medical tourism perspective. *Journal of Destination Marketing & Management*, *5*(3), 192-201. | Wrong patient population |
| 1. Abubakar, A. M., Ilkan, M., Al-Tal, R. M., & Eluwole, K. K. (2017). eWOM, revisit intention, destination trust and gender. Journal of Hospitality and Tourism Management, 31, 220-227. | Wrong patient population |
| 1. Adams, K., Snyder, J., Crooks, V., & Johnston, R. (2015). Tourism discourse and medical tourists’ motivations to travel. Tourism Review, 70(2), 85-96. | Wrong patient population |
| 1. Agbabiaka, H. I., Omisore, E. O., & Odunsi, O. (2017). Medical tourism in Nigeria: a multivariate analysis of challenges faced by patrons. International Journal of Tourism Cities, 3(4), 339-349. | Wrong patient population |
| 1. Alkelani, W., & Habil, H. (2018). The Concept of Decision-Making Among Medical Travellers: A Review of the Current State of Affairs. Advanced Science Letters, 24(6), 4358-4363. | Conference abstract |
| 1. Almodawer, Y., Alam, S. S., Sinniah, S., & Ali, M. H. (2024). Health tourism in Malaysia: understanding the drivers of satisfaction and revisit intention. Tourism Recreation Research, 1-22. | Wrong patient population |
| 1. An, D. (2014). Understanding medical tourists in Korea: Cross-cultural perceptions of medical tourism among patients from the USA, Russia, Japan, and China. Asia Pacific Journal of Tourism Research, 19(10), 1141-1169. | Wrong patient population |
| 1. Aydin, G., & Karamehmet, B. (2017). Factors affecting health tourism and international health-care facility choice. International Journal of Pharmaceutical and Healthcare Marketing, 11(1), 16-36. | Wrong patient population |
| 1. Azimi, R., Mahmoudi, G., & Esmaeili, H. A. (2018). Ranking factors affecting the attraction of foreign medical tourists in hospitals affiliated to mashhad university of medical sciences based on marketing mix model. Iranian journal of public health, 47(10), 1593. | Wrong patient population |
| 1. Bashir, U., Siddiq, G., Saleem, N., Farooq, H., Awais, M., Ussama, M., ... & Banoori, M. M. (2023). First-world care at third-world rates: Pakistan, an attractive destination for bariatric tourism. Cureus, 15(11). | Unknown patient population |
| 1. Baydeniz, E., Çılgınoğlu, H., & Valeri, M. (2024). Impact of health tourism visitor experiences on behavioral intention. Journal of Organizational Change Management, 37(5), 1133-1153. | Wrong patient population |
| 1. Boguszewicz-Kreft, M., Kuczamer-Kłopotowska, S., & Kozłowski, A. (2022). The role and importance of perceived risk in medical tourism. Applying the theory of planned behaviour. Plos one, 17(1), e0262137. | Wrong patient population |
| 1. Boguszewicz-Kreft, M., Kuczamer-Kłopotowska, S., Kozłowski, A., Ayci, A., & Abuhashesh, M. (2020). The theory of planned behaviour in medical tourism: International comparison in the young consumer segment. International journal of environmental research and public health, 17(5), 1626. | Wrong patient population |
| 1. Boguszewicz-Kreft, M., Kuczamer-Kłopotowska, S., Kozłowski, A., Ayci, A., & Abuhashesh, M. (2020Theory of Planned Behaviour in Medical Tourism. International Comparison in the Young Consumer Segment. Int Business Informat Management, Assoc. 34th International-Business-Information-Management-Association (IBIMA) Conference Nov 13-14 2019;():5023-5029. Madrid, SPAIN 2019 Nov 13-14 | Conference abstract |
| 1. Cha, J., Jo, M., Lee, T. J., & Hyun, S. S. (2024). Characteristics of market segmentation for sustainable medical tourism. International Journal of Tourism Research, 26(1), e2626. | Wrong patient population |
| 1. Cham, T. H., Cheng, B. L., Low, M. P., & Cheok, J. B. C. (2020). Brand image as the competitive edge for hospitals in medical tourism. European Business Review, 33(1). | Wrong patient population |
| 1. Cham, T. H., Lim, Y. M., Aik, N. C., & Tay, A. G. M. (2016). Antecedents of hospital brand image and the relationships with medical tourists’ behavioral intention. International journal of pharmaceutical and healthcare marketing, 10(4), 412-431. | Wrong patient population |
| 1. Cham, T. H., Lim, Y. M., Sia, B. C., Cheah, J. H., & Ting, H. (2021). Medical tourism destination image and its relationship with the intention to revisit: A study of Chinese medical tourists in Malaysia. Journal of China tourism research, 17(2), 163-191. | Wrong patient population |
| 1. Cham, T. H., Lim, Y. M., & Sigala, M. (2022). Marketing and social influences, hospital branding, and medical tourists' behavioural intention: Before‐and after‐service consumption perspective. International Journal of Tourism Research, 24(1), 140-157. | Wrong patient population |
| 1. Debata, B. R., Patnaik, B., Mahapatra, S. S., & Sree, K. (2015). Interrelations of service quality and service loyalty dimensions in medical tourism: A structural equation modelling approach. Benchmarking: An International Journal, 22(1), 18-55. | Wrong patient population |
| 1. Dryglas, D., & Lubowiecki-Vikuk, A. (2019). The attractiveness of Poland as a medical tourism destination from the perspective of German and British consumers. Entrepreneurial Business and Economics Review, 7(2). | Wrong patient population |
| 1. Dubrey, S. W. (2018). Health tourism and the NHS: a personal view. British Journal of Hospital Medicine, 79(8), 476-476. | Correspondence |
| 1. Elbaz, A. M., Abou Kamar, M. S., Onjewu, A. K. E., & Soliman, M. (2023). Evaluating the antecedents of health destination loyalty: The moderating role of destination trust and tourists’ emotions. International Journal of Hospitality & Tourism Administration, 24(1), 1-28. | Unknown patient population |
| 1. Farrukh, M., Shahzad, I. A., Sajid, M., Sheikh, M. F., & Alam, I. (2022). Revisiting the intention to travel framework in the perspective of medical tourism: The role of eWord-of-mouth and destination image. International Journal of Healthcare Management, 15(1), 28-35. | Unknown patient population |
| 1. Fengmin, Z., Baijun, W., Jiangtao, B., Li, L., & Patwary, A. K. (2022). Investigating revisit intention of medical tourists in China through nutritional knowledge, perceived medical quality, and trust in the physiologist: A recommendation on health tourism policy measures. Frontiers in Public Health, 10, 893497. | Wrong patient population |
| 1. Filippi, A., Gheorghe, R., & Zürcher, A. (2017). Dental tourism from Switzerland to Germany. SWISS DENTAL JOURNAL SSO–Science and Clinical Topics, 127(7/8), 618-633. | Wrong patient population |
| 1. Fisher, C., & Sood, K. (2014). What is driving the growth in medical tourism?. Health Marketing Quarterly, 31(3), 246-262. | Wrong patient population |
| 1. Fook, T. N., Peng, L. M., & Mun, Y. W. (2024). Hospital brand image and trust leading towards patient satisfaction: medical tourists’ behavioural intention in Malaysia. Healthcare in Low-Resource Settings, 12(1). | Wrong patient population |
| 1. Fouani, T., Minhem, M., Alami, R., Khatib, D., & Safadi, B. (2018). Bariatric medical tourism: a Lebanese center experience. In Obesity Surgery (vol. 28, pp. 255-255). 233 Spring St, New York, USA: Springer. | Conference abstract |
| 1. Ftaieh, F.; Jindal, A.; Patel, A. G.; Shanti, H. (2023). A O01 Bariatric tourism: Patients perspective. British Journal of Surgery, 110. | Conference abstract |
| 1. Gupta, H., Nigam, N., Patwa, A. K., & Kumar, S. (2021). Medical Tourism-Our Choices Decide Our Destiny. Indian Journal of Medical Specialities, 12(4), 241-242. | Correspondence |
| 1. Han, H., & Hwang, J. (2013). Multi-dimensions of the perceived benefits in a medical hotel and their roles in international travelers’ decision-making process. International Journal of Hospitality Management, 35, 100-108. | Wrong patient population |
| 1. Han, H., & Hwang, J. (2018). Growing competition in the healthcare tourism market and customer retention in medical clinics: New and experienced travellers. Current Issues in Tourism, 21(6), 680-702. | Wrong patient population |
| 1. Han, H., & Hyun, S. S. (2015). Customer retention in the medical tourism industry: Impact of quality, satisfaction, trust, and price reasonableness. Tourism management, 46, 20-29. | Wrong patient population |
| 1. Hung, Y. H., Ma, J., Chou, J. C. L., & Tzeng, G. H. (2014, September). Improving medical tourism services based on a hybrid MCDM model combined by DANP and VIKOR techniques. In 2014 IEEE International Conference on Management of Innovation and Technology (pp. 486-488). IEEE. | Wrong outcomes |
| 1. Idrus, S., Musa, R., Naziman, Y. H. N. M., Aznan, N. F. M., Othman, A. Y., & Pauzi, N. M. (2012). Medical tourism destination brand positioning model. In A. Zainal, SM Radzi, R., Hashim, CT Chik, & R. Abu,(Eds.), Current issues in hospitality and tourism research and innovations. Proceedings of the International Hospitality and Tourism Conference, IHTC (pp. 515-518). | Wrong outcomes |
| 1. Jaapar, M., Musa, G., Moghavvemi, S., & Saub, R. (2017). Dental tourism: Examining tourist profiles, motivation and satisfaction. Tourism Management, 61, 538-552. | Wrong patient population |
| 1. Jiang, M., Qiao, G., Hou, S., & Zhao, L. (2024). Understanding the impact of psychological distance on medical tourism intention: the health belief model perspective. Journal of Quality Assurance in Hospitality & Tourism, 1-25. | Wrong patient population |
| 1. Johnston, R., Crooks, V. A., & Snyder, J. (2012). “I didn’t even know what I was looking for”: A qualitative study of the decision-making processes of Canadian medical tourists. Globalization and health, 8, 1-12. | Wrong patient population |
| 1. Kim, M. (2022). A multi-level approach to perceived risks of medical tourism service and purchase intention: An empirical study from Korea. The Journal of Asian Finance, Economics and Business, 9(1), 373-385. | Wrong patient population |
| 1. Kim, M., Koo, D. W., Shin, D. J., & Lee, S. M. (2017). From servicescape to loyalty in the medical tourism industry: A medical clinic’s service perspective. INQUIRY: The Journal of Health Care Organization, Provision, and Financing, 54, 0046958017746546. | Wrong patient population |
| 1. Kim, S. M., & Um, K. H. (2016). The effects of ambivalence on behavioral intention in medical tourism. Asia Pacific Journal of Tourism Research, 21(9), 1020-1045. | Wrong patient population |
| 1. Köberlein, J., & Klingenberger, D. (2010). Foreign dentures and dental tourism--willingness-to-pay and factors influencing the demand for foreign dental prosthesis in Germany. Gesundheitswesen (Bundesverband der Arzte des Offentlichen Gesundheitsdienstes (Germany)), 73(7), e111-8. | Not English language |
| 1. Lubowiecki-Vikuk, A., & Dryglas, D. (2019). Medical tourism services and medical tourism destinations in Central and Eastern Europe-the opinion of Britons and Germans. Economic research-Ekonomska istraživanja, 32(1), 1256-1274. | Wrong outcomes |
| 1. Majeed, S., Lu, C., Majeed, M., & Shahid, M. N. (2018). Health resorts and multi-textured perceptions of international health tourists. Sustainability, 10(4), 1063. | Wrong patient population |
| 1. Manaf, N. H., Hussin, H., Jahn Kassim, P. N., Alavi, R., & Dahari, Z. (2015). Country perspective on medical tourism: the Malaysian experience. Leadership in Health Services, 28(1), 43-56. | Wrong patient population |
| 1. Manaf, N. H. A., Hussin, H., Kassim, P. N. J., Alavi, R., & Dahari, Z. (2015). Medical tourism service quality: finally some empirical findings. *Total Quality Management & Business Excellence*, *26*(9-10), 1017-1028. | Wrong patient population |
| 1. Meghani, Z. (2013). The ethics of medical tourism: From the United Kingdom to India seeking medical care. International Journal of Health Services, 43(4), 779-800. | Not primary research |
| 1. Menvielle, L., Menvielle, W., & Tournois, N. (2014). Purchasing behavior of consumers for foreign medical services: An approach using the soft laddering method. Qualitative Market Research: An International Journal, 17(3), 264-282. | Wrong outcomes |
| 1. Mohammed Abubakar, A. (2016). Does eWOM influence destination trust and travel intention: a medical tourism perspective. Economic research-Ekonomska istraživanja, 29(1), 598-611. | Wrong patient population |
| 1. Moreno-González, A. A., León, C. J., & Fernández-Hernández, C. (2020). Health destination image: The influence of public health management and well-being conditions. Journal of Destination Marketing & Management, 16, 100430. | Wrong patient population |
| 1. Na, S. A., Nee, A. Y. H., & Onn, C. Y. (2017). Medical tourism: The effects of perceived benefits, perceived risks and geographic region. Pertanika Journal of Social Sciences & Humanities, 25, 143-152. | Wrong patient population |
| 1. Noree, T., Hanefeld, J., & Smith, R. (2014). UK medical tourists in Thailand: they are not who you think they are. Globalization and health, 10, 1-7. | Wrong outcomes |
| 1. O'Halloran, A., Walsh, A., & Harrington, P. (2024). Stature seekers: Cosmetic limb lengthening in medical tourism a case report. JPRAS open, 42, 146-151. | Wrong outcomes |
| 1. Obermaier, A. J. (2009). Cross-border purchases of health services: A case study on Austria and Hungary. World Bank Policy Research Working Paper, (4825). | Wrong patient population |
| 1. Pan, T. J., & Chen, W. C. (2014). Chinese medical tourists–Their perceptions of Taiwan. Tourism Management, 44, 108-112. | Wrong patient population |
| 1. Pan, X., & Moreira, J. P. (2018). Outbound medical tourists from China: An update on motivations, deterrents, and needs. International Journal of Healthcare Management, 11(3), 217-224. | Wrong patient population |
| 1. Parmar, C., Gould, L., & Lough, M. (2017). Bariatric tourism-a single centre experience. Bariatric surgery tourism. In Obesity Surgery, 27, 434-434. 233 SPRING ST, NEW YORK, NY 10013 USA: SPRINGER. | Conference abstract |
| 1. Putit, L.; Yusof, J. M.; Khan, N. R. M.; Suki, A. A (2014) Conceptualizing Medical Tourists' Adoption of Destination Choice Behavior. 23rd International-Business-Information-Management-Association Conference on Visio 2020: Sustainable Growth, Economic Development, and Global Competitiveness, 1651-1659. Valencia, SPAIN. | Conference abstract |
| 1. Rahman, M. S., Bag, S., Hassan, H., Hossain, M. A., & Singh, R. K. (2022). Destination brand equity and tourist's revisit intention towards health tourism: an empirical study. Benchmarking: An International Journal, 29(4), 1306-1331. | Wrong patient population |
| 1. Ramamonjiarivelo, Z., Martin, D. S., & Martin, W. S. (2015). The determinants of medical tourism intentions: Applying the theory of planned behavior. Health marketing quarterly, 32(2), 165-179. | Wrong patient population |
| 1. Saiprasert, W., Xu, B., & Tavitiyaman, P. (2022). The relationships among perceived medical quality, well-being perception, and behavioral intention: A comparison between domestic and overseas medical destinations. Journal of Quality Assurance in Hospitality & Tourism, 23(4), 1011-1036. | Wrong patient population |
| 1. Saiprasert, W. (2011). Examination of the medical tourists motivational behavior and perception: A structural model. Dissertation Abstracts International Section A: Humanities and Social Sciences | Unknown patient population |
| 1. Sarhan, M., Fekry, D., & Newira, M. (2023, August). Telehealth and medical tourism in bariatric surgery with a scoping review on our experience. In Obesity Surgery (vol. 33, pp. 916-916). New York, United States: Springer. | Conference abstract |
| 1. Sarwar, A. A., Manaf, N. A., & Omar, A. (2012). Medical tourist’s perception in selecting their destination: a global perspective. Iranian Journal of Public Health, 41(8), 1. | Not primary research |
| 1. Sarwar, A., SARIF, S. M., & NIKHSHEMI, S. R. (2016). On the ethics of medical tourism: an examination of patients’ perspectives. Iranian journal of public health, 45(5), 688. | Correspondence |
| 1. Seow, A. N., Choong, Y. O., Choong, C. K., & Moorthy, K. (2022). Health tourism: behavioural intention and protection motivation theory. Tourism Review, 77(2), 376-393. | Unknown patient population |
| 1. Seow, A. N., Choong, Y. O., Moorthy, K., & Chan, L. M. (2017). Intention to visit Malaysia for medical tourism using the antecedents of Theory of Planned Behaviour: A predictive model. International Journal of Tourism Research, 19(3), 383-393. | Unknown patient population |
| 1. Shareef, M. A., Kim, D. Y., Khan, A. R., Akram, M. S., Butt, I., & Sadrul Huda, S. S. M. (2024). Understanding the behaviour of medical tourists: implications for strategy development. Journal of Policy Research in Tourism, Leisure and Events, 1-27. | Wrong patient population |
| 1. Shoukat, M. H., Elgammal, I., Aziz, S., Olya, H., & Selem, K. M. (2025). Medical tourism index and travel willingness via travel anxiety: PLS-NCA approach. Tourism Recreation Research, 50(2), 369-384. | Wrong patient population |
| 1. Snyder, J., Crooks, V. A., & Johnston, R. (2012). Perceptions of the ethics of medical tourism: comparing patient and academic perspectives. Public Health Ethics, 5(1), 38-46. | Wrong patient population |
| 1. Suki, A. A., Putit, L., Rita, N., & Khan, M. (2017). Assessing sharia compliance medical destination behaviour: A medical tourism perspective. Pertanika J Soc Sci Hum, 25, 203-14. | Wrong patient population |
| 1. Suki, A. A., Putit, L., Yusof, J. M., & Khan, N. R. M. (2014). Assessing medical tourists’ destination choice behavior: A conceptual perspective. Theory Pract. Hosp. Tour. Res, 7, 519-523. | Conference abstract |
| 1. Sukma, A. (2024). Medical tourism in central Java: Influences of image and promotion. Tourism and Hospitality Research, 14673584241311283. | Wrong patient population |
| 1. Wan, D.; Hui, T. K. (2009). Perception of Singapore's Healthcare Tourism. u Bergakadem Freiberg, Tianjin Univ Tourism Management Coll German Acad Exchange Service Fdn German Embassy Euro-Asia Conference on Environment and Corporate Social Responsibility, 101. | Conference abstract |
| 1. Wang, H., Ghasemi, M., Ghadiri Nejad, M., & Khandan, A. S. (2023). Assessing the potential growth of Iran’s hospitals with regard to the sustainable management of medical tourism. Health & Social Care in the Community, 2023(1), 8734482. | Wrong patient population |
| 1. Wang, H. Y. (2012). Value as a medical tourism driver. Managing Service Quality: An International Journal, 22(5), 465-491. | Wrong patient population |
| 1. Wang, J. H., Feng, H., & Wu, Y. (2020). Exploring key factors of medical tourism and its relation with tourism attraction and re-visit intention. Cogent social sciences, 6(1), 1746108. | Wrong patient population |
| 1. Wongkit, M., & McKercher, B. (2013). Toward a typology of medical tourists: A case study of Thailand. Tourism Management, 38, 4-12. | Wrong patient population |
| 1. Yu, J., Seo, J., & Hyun, S. S. (2021). Attributes of medical tourism destination brands: case study of the Korean medical tourism market. Journal of Travel & Tourism Marketing, 38(1), 107-121. | Wrong patient population |
| 1. Yu, J. Y., & Ko, T. G. (2012). A cross-cultural study of perceptions of medical tourism among Chinese, Japanese and Korean tourists in Korea. Tourism management, 33(1), 80-88. | Wrong patient population |
| 1. Zhong, X., & Chan, C. S. (2024). Opportunities, challenges and implications of medical tourism development in Hong Kong. International Journal of Tourism Research, 26(1), e2615. | Wrong patient population |
| 1. Ziajka, P., Waledziak, M., & Stanowski, E. (2019). How optimize costs and safety of laparoscopic procedures-life experience study bariatric surgery tourism. In Obesity Surgery (vol. 29, pp. 454-454). New York, USA: Springer. | Conference abstract |

Supplementary Material 7: Characteristics of included studies. Type of medical tourism indicates the relevant data extracted for the study aims. % female and mean and standard deviation for age are provided where the relevant data was available. ‘Mixed’ MT type refers to studies that included a range of medical tourists (that were predominantly cosmetic and/or bariatric tourists).

| **Study ID** | **Aim** | **Type of medical tourism (cosmetic, bariatric, both, mixed)** | **Setting and sample (relevant to review aims)** | **Methodology** | **Type of study and analysis** | **Main findings** | **Methodological quality** |
| --- | --- | --- | --- | --- | --- | --- | --- |
| Anar 2025 (49) | To evaluate relationships between preoperative surgical fear, anxiety,  and satisfaction in Individuals choosing bariatric surgical tourism in Turkey | Bariatric | N = 156 foreign patients who  attended a general surgery clinic of a private healthcare institution in Istanbul between July and November 2024 | Quantitative | Cross-sectional descriptive survey. | The leading factors for engaging in health tourism included higher surgical costs in home countries, recommendations from previously satisfied individuals, prolonged waiting times for surgery, and failure to meet the eligibility criteria for bariatric surgery in their own countries.  Age, fear and anxiety associated with undergoing surgery in a foreign country were independent predictors of patients’ satisfaction with their bariatric surgery tourism experience | Potential lack of representativeness of the sample due to the study being undertaken in a single clinic and non-response bias |
| Arrobas 2021 (50) | To investigate if Lisbon is an economical destination for English MTs and to measure which motivational factors affect their selection of a destination | Cosmetic (dental) | N = 346 English MTs aged at least 30 (M = 51.9 (SD = 14.5, 55.8% women) and earning the at least the average income in the UK, stratified sampling based on age, gender, and household income | Quantitative | Cross-sectional questionnaire. willingness to pay, EFA, and CFA. | Lisbon is within remit as a MT destination for English tourists based on price and distance. The main concerns of MTs traveling abroad for MT related to  “Quality, Safety and Affordability”.  Past uptake of MT was associated with future MT intent. | Potential lack of representativeness of the sample due to non-response bias |
| Baan, 2023 (37) | To understand how MTs search online and why they use social media when planning and organizing their trips | Cosmetic | N=25 participants (from generation Y or Z (born between 1980-2005), with exposure to social media, 52% female) from 19 countries, convenience and snowball sampling through online via social media platforms | Qualitative | Semi-structured interviews. Leximancer (a software that detects trends and connections within text). | The following six key themes were identified that explained influencers' impact on the intent to engage in medical tourism: procedures, social, information, surgery, money and place | Reductive data analysis and no supporting data (quotes). |
| Carmagnola 2012 (51) | To investigate the perception of dental tourism by Italian MTs | Cosmetic (dental) | N = 81 participants who were members of an Italian consumers organisation | Quantitative | Cross-sectional descriptive survey. | Reasons for participating in MT included (lower) cost, advice from friends/relatives, trust in foreign dentists, other reasons (e.g., had a foreign spouse, had a toothache whilst travelling abroad, had bad experiences with Italian dentists). Specific dental practices were chosen based on recommendations of friends or relatives, advertisements, found or knew of the dentists. | Basic, un-piloted questionnaire with pre-defined reasons for MT for participants to select from. Lack of information re representativeness of the sample. |
| Chia 2021 (38) | To explore the factors influencing Chinese mainland outbound MT | Mixed | N = 10 prospective Chinese MTs (data from 2 participants was extracted), recruited via purposive sampling through the researcher’s network | Qualitative | Semi-structured telephone interviews. Thematic analysis. | Quality of treatment, Quality of customer service, Cost and Travel distance were factors that influenced Chinese outbound MT | Interview guide not provided and analysis methods not described in detail |
| Hanefeld 2015 (39) | To identify factors influencing decision to participate in MT and the choice of destination | Mixed | N=77 MTs from the UK, recruited through a variety of sources (e.g., adverts, posts on forums) | Qualitative | In-depth interviews and focus groups. Thematic analysis using framework method. | Motivations for travel were availability (e.g., patients ineligible for treatment on the NHS), cost (MT cheaper than private treatment in the UK), expertise of the clinician, cultural/ family (e.g., ability to speak a certain language) or recommendations/ word of mouth/ online groups.  Comparison across types of MT: cosmetic and bariatric tourism was particularly bound to value for money. Cost a particular priority for cosmetic surgery, whilst availability, experience of the surgeon, proximity of destination country (for postoperative travel) was particularly important for bariatric surgery. Bariatric tourists particularly valued support groups and noted that surgeons would infiltrate support groups with advertisements. | Insufficient provision of participant quotes |
| Holliday 2014 (40) | To understand MT experiences, the organisations involved, and the implications for globalized healthcare | Mixed | N= 103 MTs from the UK, Australia and China; recruitment methods not described | Qualitative | Interviews, observations, photo diaries, video diaries, online questionnaire. Thematic analysis. | Factors influencing MT were cost, surgical quality, technique and technology. Reasons for surgery were correction of perceived ‘abnormalities’, investment (e.g., trying to look fashionable), ‘repair’ (e.g., post-pregnancy surgery) and anti-ageing. The quality of the surgeon as their primary reason for choosing a specific destination. The clinic and destination country was of secondary importance, although these often followed cheap flights.  Information sources: attributed the internet as key, including provider websites, social media where providers often advertised, and social networking sites for peer support. Over half of MTs utilised an agent to arrange their trip. | Lack of detail and therefore transparency around the methods |
| Jackson 2018 (41) | To better understand the motivating factors that are driving the practice of bariatric tourism by Canadians | Bariatric | N=20 mostly female former Canadian bariatric MTs who travelled to Mexico, recruited online using snowball sampling | Qualitative | Semi-structured interviews. Thematic analysis. | The following barriers to accessing care domestically were the main motivations for travelling abroad: Not meeting the body mass index (BMI) requirements; Structural barriers to accessing surgery domestically e.g. concerns about the quality locally) and lengthy wait times | Insufficient detail around analysis (specifically around how conflicting data was handled) |
| Jackson 2019 (42) | To explore Canadian bariatric patients’ experiences of seeking and obtaining weight loss surgery abroad | Bariatric | Same sample as above | Qualitative | Semi-structured interviews. Thematic analysis. | Stigma of being overweight led participants to not inform family and friends of their decision to undergo bariatric surgery. Bariatric surgery wasn’t presented as a viable option by healthcare professional in participants home country. This led participants to seek information themselves, primarily online, from former bariatric tourists, which enforced their desire to go abroad. | Same as above. Participant quotes not labelled. |
| Jones 2016 (48) | To explore how cosmetic MTs use social media to conduct research and then to navigate, document and narrate their experiences | Cosmetic | Same sample as Holliday, 2014 | Qualitative | Interviews, observations, photo diaries, video diaries, online questionnaire. Thematic analysis. | Cosmetic surgery tourism is a ‘buyer beware’ market where the client is increasingly seen as being responsible for knowing risks and for making the ‘right’ decisions (in relation to technologies, surgeons, hospitals, countries and products etc.).  Participants seek information about MT online; used a range of sources and felt that they had conducted sufficient research. Testimonials and stories of previous recipients are often of most value and most trusted to those in the anticipatory phases of their journeys. Accessing others' stories allowed participants to negotiate the various possibilities of cosmetic surgery tourism. | NA |
| Kim 2016 (17) | To estimate costs of treating complications related to MT in bariatric surgery and to understand patients’ motivations for pursuing MT | Bariatric | N = 14 postoperative Canadian MTs from Alberta, recruited via two web forums | Quantitative | Cross sectional descriptive survey | Common reasons for seeking bariatric surgery via medical tourism included long wait lists and presumed ineligibility for domestic surgery.  Most believed their bariatric surgeries were successful despite some experiencing postoperative complications. The most common MT destination was Mexico followed by the USA. | No details of the survey, response rate, or sampling strategy are provided |
| Lee 2012 (52) | To identify salient beliefs items (i.e. behavioural beliefs, normative beliefs, subjective norms, and control beliefs) for each ToPB predictor construct for each model that assessed intention to travel to Korea for either health treatment (HT) (medically necessary operations e.g. heart surgery, hip replacement) or beautification treatment (BT) (e.g. face lift, tummy tuck, wrinkle removal) | Cosmetic | N = 237 Japanese MTs (71.7% female) recruited at entry/exit points (ferry terminal, airport) in Korea. | Quantitative | Cross sectional survey. CFA and SEM. | The ToPB for both models exerted a reasonable fit and showed a reasonable predictive power for intention (explaining 28% and 31% of the variance in intention for the HT and BT models, respectively). All paths were positive and significant. For the BT model, the effect of subjective norms was inferior compared to the effects of attitude and perceived behaviour control (the opposite was observed for the HT model). | No details of the response rate or representativeness of the sample |
| Liang 2019 (53) | To apply Martin et al.'s (2011) 29‐item MEDTOUR scale and add the antecedents of perceived risk and perceived benefit to predict the intention of Chinese adults to travel oversees for cosmetic treatments | Cosmetic | N = 522 Chinese adults (83.7% female, mostly young: M = 30.3) considering cosmetic tourism, recruited at cosmetic surgery hospitals in China | Quantitative | Cross sectional survey. CFA and SEM. | The MEDTOUR  scale achieved acceptable level of factorial, convergent, and discriminant validity.  The model exerted an acceptable fit and all hypothesised relationships between constructs were significantly supported; perceived risk negatively predicted and perceived benefit positively predicted; attitude, perceived behavioural control and subjective norm, which all predicted intention. In addition, attitude mediated the relationship between perceived behavioural control and subjective norm. | No details of the survey are provided |
| Majeed 2020 (54) | To explore tourists’ perceptions  of cosmetic tourism for medical service quality as an antecedent to tourists’ emotional attachment, trust, and intentions to visit | Cosmetic | N = 279 (57% female) Australian, Japanese, and Chinese international tourists considering cosmetic tourism, recruited from 2 airports in China | Quantitative | Cross sectional survey. Partial least-square SEM. | Perceived medical service quality significantly predicted emotional attachment to cosmetic tourism services, trust in cosmetic tourism, and intentions to engage in cosmetic tourism, partially mediated by value co-creation (interacting with the provider to meet their expectations and agree on treatment together). | No details on representativeness of the sample or response rate, concerns around validity of the survey items |
| Majeed 2024 (59) | To examine the underlying mechanism of cognitive dissonance in MT and its impact on tourist behaviours. Study 1 (only study extracted): to identify the factors that induce tourists to make pre-purchase decisions regarding MT and determine the hidden mechanism of cognitive dissonance | Mixed | N = 26 (42.3% female) postoperative Chinese MTs recruited through an online forum and medical travel agents | Mixed | Semi-structured interviews. Used the laddering technique. | Decision making factors of MT vary on the type of MT; in a non-critical disease condition, medical tourists expect a medical tourism package that offers regular or ordinary medical assistance, such as a health checkup and non-surgical treatment without an inpatient stay, and more visits to tourist attractions.  MTs identified different criteria for choosing their Mt package e.g. to do with the surgeon, cost, destination, or accommodation. Perceived risk influenced how much MT’s wanted to combine their treatment a with vacation, and their intentions in engaging in MT. | Many concerns including lack of explanation for use of qualitative data, lack of detail around data collection and analysis of both data sources, and lack of triangulation |
| Martin 2019 (55) | To review the surge in patients admitted with complications in the Plastic Surgery Unit following cosmetic surgery abroad. Specifically, to review the perioperative pathway abroad, the driving forces behind seeking out surgery abroad, and the financial impact this has on the NHS. | Cosmetic | N = 6 (all) patients admitted to the unit in Northern Ireland following cosmetic tourism, all of whom had received multiple procedures abroad | Quantitative | Cross sectional descriptive survey | Reasons for seeking out surgery abroad included family or friend recommendation, a perceived cheaper cost, convenience, and access to procedures that were not recommended or advised against by a UK surgeon | Lack of details of the survey, very small sample with limited generalisability, lack of reporting of findings |
| Mutalib 2017 (43) | To explore the written online narratives about MT, to develop an understanding of the current needs and gaps in  terms of services given and perceived satisfaction levels  of MTs | Mixed | N = 43 posters (MTs or their caretakers, mostly from Australia) discussing MT in Thailand or Malaysia, collected from blog posts and forums | Qualitative | Publicly available online narratives. Content analysis. | Push factors including high cost of treatment in home country (which was often the main motivation) and inadequate insurance in home country.  Most posters discussed pull factors, including cheap cost of travel and treatment (which was the main reason for destination choice for all posters), availability of facilitators or familiarity/ friends and family in the destination country, local attractions, a bandwagon effect, hearing success stories, and the expertise of surgeons. | Lack of clarity around research aims, analysis method not described in detail, and insufficient provision of participant quotes |
| Nam 2020 (56) | To develop a valid and reliable scale to measure the perceived risk of cosmetic surgery tourism, then to segment the cosmetic surgery tourism market based on this, to identify the personal and behavioural characteristics of the different segments | Cosmetic | N = 1047 prospective Chinese cosmetic surgery tourists (74% women) interested in or planning to have cosmetic surgery in South Korea, recruited via a research agency | Quantitative | Cross-sectional survey. EFA, CFA, and segmentation using latent class modelling and the chi-square automatic interaction detection (CHAID) algorithm | Risks around time and cost (Cost risk) had highest mean scores, compared with medical (poor surgical outcomes or poor performance of medical service providers), vacation (unfavourable situations encountered by cosmetic surgery tourists after their cosmetic procedures, such as complications, insufficient vacation opportunities, and immigration issues), and destination risks (the hostile environment of a cosmetic surgery tourism destination).  Segmentation identified three groups; risk neutral (did not perceive any risks, 39% of the sample, wanted to spend the most money on surgery with focus on facelift/contouring), risk concerned (perceived cost and vacation risks, but not medical and destination risk, 39% of the sample), and risk sensitive (scored higher on all four types of risk, 22% of the population, less interested in invasive or high risk cosmetic procedures) | Insufficient details of recruitment sources and representativeness of the sample |
| Nassab 2010 (57) | To investigate public opinion on undergoing cosmetic surgery abroad and then explore the information patients are likely to encounter on the Internet when searching for such services | Cosmetic | N = 197 adults from the UK, recruited from a retail centre (98.5% response rate) | Quantitative | Cross-sectional descriptive survey. | Of those who had considered cosmetic surgery, almost all would consider surgery abroad.  Of these, the most common motivation was cost, and the most accessed information source were the internet.  Most responded that they would contact their local health service if complications arose. | Survey items not provided, concerns around relevance and representativeness of recruitment source |
| Noaman 2023 (44) | To determine the factors influencing cosmetic tourists’ choices of their destination, cosmetic doctor, and medical center | Cosmetic | N = 18 cosmetic tourists and medical doctors (extracted 4 relevant participants), purposively sampled from cosmetic doctors in Lebanon after surgery | Qualitative | Interviews. Thematic analysis. | Important to cosmetic tourists’ decision was suitability of the surgery result to the ethnicity of MTs, hygiene, and information from relatives.  MTs discussed problems around lack of clarity around cost and variable quality of care. | Lack of coherence and explanation of data analysis |
| Ozan-Rafferty 2014 (45) | Aimed to identify characteristics of MTs that travel to Turkey, leading push and pull factors, satisfaction, and influences of perceptions about travelling to Turkey for MT | Mixed | N = 36 MTs or their significant other or partner discussing their experience of surgery in Turkey, purposively sampled | Qualitative | Publicly available online narratives. Narrative analysis. | Most common push factors were total costs, lack of insurance coverage, and lack of treatment options in MT’s home country.  Most common pull factors were cost and value for money, expertise of the surgeon, availability of a facilitator, communication and responsiveness of providers, and familiarity or interest in Turkey.  Sources of support and information included use of a MT facilitator, connecting with peers, and internet research. | Lack of supporting data (quotes) provided |
| Prasad 2024 (46) | To conduct an in-depth analysis of the significance of each information resource in the decision-making process of cosmetic tourists | Cosmetic | N = 8 Australian prospective or previous cosmetic tourists (mainly female), recruited online and through the university | Qualitative | Semi-structured interviews. Reflexive thematic analysis | Themes:  Long term decision to undergo cosmetic procedures  Importance of different information resources: Websites, Existing social network, Online support communities, and Surgeon consultations | None |
| Robertson 2022 (47) | To review Canadians’ experiences with travelling abroad for cosmetic surgery, including primary motivations for seeking care outside of Canada | Cosmetic | N 11 Canadian cosmetic tourists who required referral to a domestic plastic surgeon on their return, recruited at plastic surgeon offices in Canada | Qualitative | Semi structured interviews. Thematic analysis. | Motivational for MT were cost, availability of post-operative care, marketing/ customer service, word-of-mouth, concerns with local surgeon, availability, family, and cleanliness.  Most common information sources were pamphlets from the provider’s office, communication with an office contact, and visiting the provider’s website, while social media, personal research, and communicating with a nurse were mentioned only one time each.  Patients discussed a lack of explanation of the risk of complications prior to surgery, and a lack of help from providers on experiencing complications. | Insufficient reporting of methods (interview guide and analysis method) and minimal interpretation and supporting data (quotes) |
| Thayarnsin 2023 (60) | To explore the relationships between risk, image, quality, satisfaction and consumer loyalty in the context of MT | Mixed | N = 205 (survey, 65.4% female) and 13 (interviews, 92% female) MTs in Thailand, collected through convenience sampling at clinics in Thailand | Mixed methods | Semi structured interviews and cross-sectional survey. Miles and Huberman's (1994) model of  qualitative data analysis and SEM. | Qualitative themes were:  Risk or fear before traveling to Thailand for medical treatment  Risk or fear during staying in Thailand for medical treatment  Risk or fear after traveling to Thailand for medical tourism  Risk affecting customer loyalty toward Thailand as a medical tourism destination  Perceived risk predicted affective image but not cognitive image. Both cognitive and affective image predicted overall image of destination. Perceived quality significantly predicted overall image of destination and satisfaction. Overall image of destination and satisfaction predicted loyalty. Perceived risk partially moderated the relationship between satisfaction and loyalty. Satisfaction positively mediated the relationship between quality and loyalty. | Many concerns including lack of explanation for use of qualitative data, lack of detail around data collection and analysis of the quantitative data, and lack of triangulation |
| Whiteman 2025 (58) | To establish patient motivations for MT | Cosmetic | N = 7 (only relevant data extracted) cosmetic MTs from the UK presenting with complications in a domestic hospital | Quantitative | Cross-sectional descriptive survey | Cost was the most common motivating factor for cometic tourism, as all felt that surgery would be cheaper than in the UK | Insufficient details of response rate, very small sample with limited generalisability, concerns around validity of survey items |

Abbreviations: MT- medical tourism, MTs – medical tourists, EFA- exploratory factor analysis, CFA – confirmatory factor analysis, SEM – structural equation modelling, ToPB – Theory of Planned Behaviour

Supplementary Material 8: Supporting data for the narrative synthesis.

Supplementary Material 7a: descriptive statistics

| **Study ID** | **Main findings** |
| --- | --- |
| **Bariatric** | |
| Retrospective | |
| Anar 2015 | The most common reasons for MT (multiple choice) were; Higher costs of surgery in the country of residence (88.5), Recommendation by previously satisfied people (53.2), Waiting time for surgery in the country of residence is too long (39.1), Inability to meet the criteria for bariatric surgery in the country of residence n = 45 (28.8), and Expect to have a better service n = 38 (24.4)  Most patients had communicated with at least one member of the surgical team before the surgery (83.3%)  Most had knowledge about bariatric surgical tourism before the procedure (53.2%) |
| Kim 2016 | Most common reasons for MT included (multiple choice); long wait lists (78.6%) and presumed ineligibility for surgery in home country (42.9%)  92.9% believed their surgery was successful despite 23.1% of those participants experiencing complications |
| **Cosmetic** | |
| Hypothetical/intentions | |
| Arrobas 2021 | The main concerns of MTs traveling abroad for MT related to “Quality, Safety and Affordability”.  Most would want to travel on their own, without a family member (70.1%). There was more variability in people who had been before (37.2%) or were planning to go (54.3%). |
| Nam 2020 (already intending or planning on engaging in MT) | Most (44%) would stay for 1-2 weeks, most (54% would travel with family and friends although 30% would go with someone who would also undergo cosmetic surgery  Most would stay in a hotel (63%) instead of a specialised accommodation for postoperative recovery.  Most (49%) would travel equally for cosmetic surgery and vacation purposes, range of budgets for expenditure.  Most of the respondents said that they would choose their cosmetic surgery clinic (86%) and type of cosmetic surgery procedure (83%) before departure.  Time and financial risks (Cost risk) had highest mean scores, compared with medical (represented the problems related to poor surgical outcomes or poor performance of medical service providers), vacation (unfavorable situations encountered by cosmetic surgery tourists after their cosmetic procedures, such as complications, insufficient vacation opportunities, and immigration issues), and destination risks (the hostile environment of a cosmetic surgery tourism destination). |
| Nassab 2010 | 70% of people interested in cosmetic tourism had accessed the Internet as a source of information when researching cosmetic surgery  Among those who would consider surgery abroad, the most frequent reason given was lower costs (88.3%). Almost one-fifth (18.1%) said they would consider surgery abroad because it would also be a vacation opportunity.  When asked whom they would contact if a complication should arise, 61.4% stated they would see their local health service. Only 36.5% said that they would contact the operating surgeon. |
| Retrospective | |
| Carmagnola 2012 | The most common reasons for MT were (multiple choice); To save money (72.8%), Advised by friends/relatives (46.9%), Trust in foreign dentists (n = 13.6), Different reasons (30.9%) e.g. had a foreign wife or husband, had a toothache whilst travelling abroad, had bad experiences with Italian dentists, felt cost was unjustified (assumed same quality of care across countries), or a shorter rehabilitation period.  The most common reasons for provider choice were; Choice of dental practice abroad, Following the recommendations of friends or relatives (66.6%), Found or knew of the dentists themselves (21%), Chose through advertisements on the web (12.4%) |
| Martin 2019 COMPLICATIONS | The most common reasons for MT were; family or friend recommendation, a perceived cheaper cost, convenience, and access to procedures that were not recommended or advised against by a UK surgeon |
| Robertson 2022 COMPLICATIONS | The most common reasons for MT were; cost (82%), postoperative care (73%), marketing/customer service (73%), and word-of-mouth (73%), and concern regarding the quality of care delivered by local surgeons (45%)  The most common sources of information were; pamphlets from the office (55%) and communication with an office contact (55%). Two patients indicated that they directly visited the website to gather preoperative information (18%), while social media, personal research, and communicating with a nurse were mentioned only one time each (9%) |
| Whiteman 2025  COMPLICATIONS | Of 5 MTs, the most common reasons for MT was (single choice); Cost (80%), only one said recommendation (20%)  Of all MTs that the question was relevant to, 100% felt that their procedure would be cheaper than in the UK. |

Supplementary Material 7b: inferential statistics

| **Study ID** | **Main findings (p values where appropriate)** |
| --- | --- |
| **Cosmetic** | |
| Hypothetical/intentions | |
| Arrobas 2021 | Past uptake of MT was associated with future MT intent (p<0,001)  Those planning to engage in MT were prepared to travel significantly further (p<0,001) and pay significantly more for accommodation (p<0,001) than those who were not, but there were no significant differences in willingness to pay for airfare (p = 0.246) or treatment (p = 0.131). |
| Lee 2012 | Tested the application of the theory of planned behaviour. Both models (health (p < .001) and beautification (p < .001) treatment) had good fit using confirmatory factor analysis, and adequately predicted the Japanese tourists’ intention to travel to Korea for medical tourism using SEM, explaining 28% (health model) and 31% (beatification model) of the variance in intention. All paths between constructs were positive and significant (p < .01 in all instances) in both models. Comparatively to travelling for health treatment, intentions to travel for beautification treatment was more influenced by attitude and perceived behavioural control than social norms (opinions, recommendations, and words of important referent groups), which was the least significant predictor. |
| Liang 2019 (already planning on engaging in MT) | Tested the application of the theory of planned behaviour, with the addition of perceived risk and perceived benefit as predictors of attitude, perceived behavioural control, and subjective norm. All paths between constructs were positive and significant: perceived risk significantly predicted attitude (p = .003), perceived behavioural control (p < .001), and subjective norm (p < .001), and perceived benefit significantly predicted attitude, perceived behavioural control, and subjective norm (p < .001 in all instances). In turn, attitude (p < .001), perceived behavioural control (p = .007), and subjective norm (p < .001) significantly predicted intention. |
| Majeed 2020 (interested in engaging with CT) | Value co-creation (interacting with the provider to meet their expectations and agree on treatment together) partially mediated effects of medical service quality on emotional attachment to CT services, tourists’ trust to avail themselves of CT services, and tourists’ intentions to visit a CT destination. All significant at the .05 significance level. |
| Nam 2020 (already intending or planning on engaging in MT) | Segmentation identified three groups; risk neutral (did not perceive any risks, 39% of the sample, wanted to spend the most money on surgery with focus on facelift/contouring), risk concerned (perceived cost and vacation risks, but not medical and destination risk, 39% of the sample), and risk sensitive (scored higher on all four types of risk, 22% of the population, less interested in invasive or high risk cosmetic procedures) |
| **Mixed** | |
| Retrospective | |
| Thayarnsin 2023 | Perceived risk predicted affective image but not cognitive image. Both cognitive and affective image predicted overall image of destination. Perceived quality significantly predicted overall image of destination and satisfaction. Overall image of destination and satisfaction predicted loyalty. Perceived risk partially moderated the relationship between satisfaction and loyalty (p>0.01).  Satisfaction positively mediates the relationship between quality and loyalty (p<0.01). |

Supplementary Material 9: Narrative synthesis of included quantitative studies in accordance with the SwiM guidance.

Bariatric tourism

Only two included quantitative studies explored bariatric tourism (Anar & Kırtıl, 2025; Kim et al., 2016). Both provided very minimal descriptive data of relevance to the research question. Amongst the most common reasons for MT identified across both studies were (assumed or actual) ineligibility for care and long waiting times in MTs’ home countries. One study identified cost as the most common reason followed by recommendation by a previous patient, with almost one quarter of participants noting that they travelled abroad for an improved service (Anar & Kırtıl, 2025). Of the available data on information seeking behaviour, most (83%) participants had communicated with the surgical team and had knowledge about bariatric tourism before the surgery (52%) (Anar & Kırtıl, 2025). Despite experiencing complications, three respondents still believed that their bariatric surgery was successful (Kim et al., 2016).

Cosmetic tourism

Most included quantitative studies (n = 10) explored cosmetic surgery tourism and could be divided into four groups; hypothetical studies either examining the intent of the general population (Arrobas, 2021; Nassab et al., 2010) or with those intending on or planning on engaging in MT (Nam, 2020), and retrospective studies either sampling the general MT population (Carmagnola et al., 2012) or focusing on those who experienced complications (Martin et al., 2019; Robertson, Moorman, & Korus, 2022; Whiteman et al., 2025). Of the descriptive data, a reduced cost was identified as the most common (Carmagnola et al., 2012; Nassab et al., 2010; Robertson, Moorman, & Korus, 2022; Whiteman et al., 2025), or one of the most common (Martin et al., 2019), reason for seeking surgery abroad. Other common reasons included diasporic MT, recommendation from a friend or family member (Carmagnola et al., 2012; Martin et al., 2019; Whiteman et al., 2025), word of mouth, access to procedures not available in the MT’s home country, perceived higher quality of care (Arrobas, 2021; Robertson, Moorman, & Korus, 2022), availability of postoperative care, and perceived improved customer service (Majeed, Zhou, & Ramkissoon, 2020; Robertson, Moorman, & Korus, 2022). Recommendation from a family or friend also influenced specific choice of provider (Carmagnola et al., 2012), and many MTs were accompanied by them for the surgery (Nam, 2020), with a higher proportion of participants stating that they would not go alone if they intended to engage in MT in comparison with those not intending to engage in MT (Arrobas, 2021). MTs who experienced complications identified access to procedures that were denied or advised against by UK surgeons as one of the most common reasons for MT (Martin et al., 2019), and appealing advertisements and postoperative care to be one of the most common motivating factors (Robertson, Moorman, & Korus, 2022). Whilst hypothetical MTs mostly accessed the internet (Nassab et al., 2010), one study of MTs who experienced complications identified information sources supplied by the provider, such as pamphlets or communications with the surgeon’s office, as the most common source of information when decision making (Robertson, Moorman, & Korus, 2022). Across those who had not yet engaged in MT, an opportunity for tourism and vacation could also be a motivating factor (Nam, 2020; Nassab et al., 2010).

Of the data that produced inferential statistics, five studies reported on cosmetic tourism (Arrobas, 2021; Lee, Han, & Lockyer, 2012; Liang et al., 2019; Majeed, Zhou, & Ramkissoon, 2020; Nam, 2020), divisible into samples of the general population (Arrobas, 2021; Lee, Han, & Lockyer, 2012) and those that specifically focused on participants who intended or planned on engaging in MT (Liang et al., 2019; Majeed, Zhou, & Ramkissoon, 2020; Nam, 2020). Studies across both sample types identified the Theory of Planned Behaviour (TPB) to exert a good fit on the data, and found that all three constructs (attitude, perceived behavioural control, and subjective norm) significantly predicted intention (Lee, Han, & Lockyer, 2012; Liang et al., 2019), explaining 31% of its variance (Lee, Han, & Lockyer, 2012). Additionally, one study of Japanese tourists travelling toe Korea identified social norms as the least influential construct on behaviour. By contrast, the authors also applied the same model to intentions to engage in MT related to health treatment and found social norms to be the strongest predictor of intention (Lee, Han, & Lockyer, 2012). Thus, opinions of friends and family appear to be less important in determining intention if the target surgery is cometic rather than health focused. Also, a study of participants who intended on engaging in cosmetic tourism identified both perceived risk and perceived benefit to be significant predictors of all three constructs (Attitude, Perceived behavioural control, and Subjective norm) (Liang et al., 2019). Additionally, one study found past uptake of MT to significantly predict intention to engage in MT (p < .001) (Arrobas, 2021), and another identified perceived quality to be a significant predictor of intention to engage in MT, which was partially mediated by interaction with the service provider to agree on treatment that added value to both parties (described as ‘value co-creation’) (Majeed, Zhou, & Ramkissoon, 2020). Another study applied segmentation of participants who intended to engage in cosmetic tourism and identified three distinct groups (Nam, 2020). The most common two groups either demonstrated little concern for risks (39%) or only were concerned about risks relating to time and costs and receiving surgery abroad (39%) (Nam, 2020). The former group were willing to spend a large amount of money on cosmetic tourism and centred around intentions to receive facelifts and contouring (Nam, 2020). Only 22% of the sample demonstrated concern for all four types of risk identified (Nam, 2020). Across groups, the largest concern surrounding risks were around time and financial risks (Nam, 2020).

Mixed

One inferential study included a mixed eligibility sample (both in terms of their eligibility based on origin country and type of MT) which supported the above finding that past behaviour predicted future behaviour, negatively moderated by perceived risk (Thayarnsin, 2023). Also, like the findings above that identified perceived quality of care as a predictor, perceived quality of care was found to impact likelihood of returning to a particular provider, partly mediated by satisfaction (Thayarnsin, 2023).

References

Anar, E. N., & Kırtıl, İ. (2025). Relationship Between Preoperative Surgical Fear, Anxiety, and Satisfaction Levels in Individuals Choosing Bariatric Surgery Tourism: A Descriptive, Cross-Sectional Study. *Obesity Surgery*, 1-11.

Arrobas, F. (2021). Dental tourism: How to promote Lisbon as a destination for the English population?

Carmagnola, D., Filippucci, L., Celestino, S., Carrassi, A., Delia, S., & Lodi, G. (2012). A survey on the experience with dental tourism in a sample of Italian patients. *Minerva Stomatologica*, *61*(1-2), 11-20. <https://ovidsp.ovid.com/ovidweb.cgi?T=JS&CSC=Y&NEWS=N&PAGE=fulltext&D=med9&AN=22274306>

Kim, D. H., Sheppard, C. E., de Gara, C. J., Karmali, S., & Birch, D. W. (2016). Financial costs and patients' perceptions of medical tourism in bariatric surgery. *Canadian Journal of Surgery*, *59*(1), 59-61. <https://doi.org/10.1503/cjs.004215>

Lee, M., Han, H., & Lockyer, T. (2012). MEDICAL TOURISM-ATTRACTING JAPANESE TOURISTS FOR MEDICAL TOURISM EXPERIENCE. *Journal of Travel & Tourism Marketing*, *29*(1), 69-86. <https://doi.org/10.1080/10548408.2012.638564>

Liang, L. J., Choi, H. C., Joppe, M., & Lee, W. (2019). Examining medical tourists' intention to visit a tourist destination: Application of an extended MEDTOUR scale in a cosmetic tourism context. *International Journal of Tourism Research*, *21*(6), 772-784. <https://doi.org/10.1002/jtr.2303>

Majeed, S., Zhou, Z. M., & Ramkissoon, H. (2020). Beauty and Elegance: Value Co-Creation in Cosmetic Surgery Tourism. *Sage Open*, *10*(2). <https://doi.org/10.1177/2158244020932538>

Martin, S., Long, R., Hill, C., & Sinclair, S. (2019). Cosmetic Tourism in Northern Ireland. *Annals of Plastic Surgery*, *83*(6), 618-621. <https://doi.org/10.1097/sap.0000000000002081>

Nam, H.-m. (2020). Perceived risk of cosmetic surgery tourism: scale development and its application in segmenting Chinese cosmetic surgery tourists.

Nassab, R., Hamnett, N., Nelson, K., Kaur, S., Greensill, B., Dhital, S., & Juma, A. (2010). Cosmetic tourism: public opinion and analysis of information and content available on the Internet. *Aesthetic Surgery Journal*, *30*(3), 465-469.

Robertson, E. M., Moorman, S. W. J., & Korus, L. J. (2022). Why Do Canadians Travel Abroad for Cosmetic Surgery? A Qualitative Analysis on Motivations for Cosmetic Surgery Tourism. *Plastic Surgery*, *30*(4), 353-359. <https://doi.org/10.1177/22925503211019607>

Thayarnsin, S. L.-o.-i. (2023). The role of risk, culture, image and quality on destination loyalty: Perspectives from international medical tourists toward Thailand as a medical tourism destinationole of risk, culture, image and quality on destination loyalty: Perspectives from international medical tourists toward Thailand as a medical tourism destination. *Dissertation Abstracts International: Section B: The Sciences and Engineering*, *84*(8-B), No Pagination Specified. <https://ovidsp.ovid.com/ovidweb.cgi?T=JS&CSC=Y&NEWS=N&PAGE=fulltext&D=psyc22&AN=2023-55666-012>

Whiteman, E., Romain, K., Welman, T., Mitchell, C., Gabuniya, N., Collins, D., & Markeson, D. (2025). The rising NHS burden from cosmetic surgery procedures performed abroad and non-surgical procedures performed in the United Kingdom. *Journal of Plastic Reconstructive and Aesthetic Surgery*, *102*, 39-41. <https://doi.org/10.1016/j.bjps.2025.01.017>
